# Sustainable Drug Discovery of Multi-Target-Directed Ligands for Alzheimer’s Disease

**DOI:** 10.1021/acs.jmedchem.1c00048

**Published:** 2021-04-08

**Authors:** Michele Rossi, Michela Freschi, Luciana de Camargo Nascente, Alessandra Salerno, Sarah de Melo Viana Teixeira, Florian Nachon, Fabien Chantegreil, Ondrej Soukup, Lukáš Prchal, Marco Malaguti, Christian Bergamini, Manuela Bartolini, Cristina Angeloni, Silvana Hrelia, Luiz Antonio Soares Romeiro, Maria Laura Bolognesi

**Affiliations:** † Department of Pharmacy and Biotechnology, Alma Mater Studiorum - University of Bologna, Via Belmeloro 6, 40126 Bologna, Italy; ‡ Department for Life Quality Studies, Alma Mater Studiorum - University of Bologna, Corso d’Augusto 237, 47921 Rimini, Italy; § Department of Pharmacy, Health Sciences Faculty, University of Brasília, Campus Universitário Darcy Ribeiro, 70910-900 Brasília, DF, Brazil; ∥ Département de Toxicologie et Risques Chimiques, Institut de Recherche Biomédicale des Armées, 91220 Brétigny-sur-Orge, France; ⊥ Biomedical Research Center, University Hospital, Sokolska 581, 500 05 Hradec Kralove, Czech Republic; # Department of Toxicology and Military Pharmacy, Faculty of Military Health Sciences, University of Defence, Trebesska 1575, 500 01 Hradec Kralove, Czech Republic; ∇ School of Pharmacy, University of Camerino, Via Madonna delle Carceri 9, 62032 Camerino, MC, Italy

## Abstract

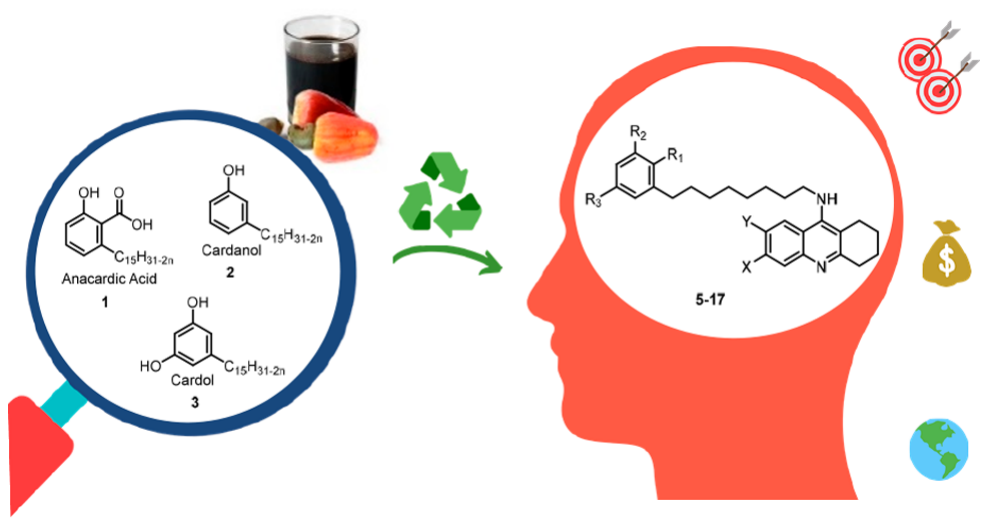

The multifactorial nature of Alzheimer’s disease (AD) is a reason for the lack of effective drugs as well as a basis for the development of “multi-target-directed ligands” (MTDLs). As cases increase in developing countries, there is a need of new drugs that are not only effective but also accessible. With this motivation, we report the first sustainable MTDLs, derived from cashew nutshell liquid (CNSL), an inexpensive food waste with anti-inflammatory properties. We applied a framework combination of functionalized CNSL components and well-established acetylcholinesterase (AChE)/butyrylcholinesterase (BChE) tacrine templates. MTDLs were selected based on hepatic, neuronal, and microglial cell toxicity. Enzymatic studies disclosed potent and selective AChE/BChE inhibitors (**5**, **6**, and **12**), with subnanomolar activities. The X-ray crystal structure of **5** complexed with BChE allowed rationalizing the observed activity (0.0352 nM). Investigation in BV-2 microglial cells revealed antineuroinflammatory and neuroprotective activities for **5** and **6** (already at 0.01 μM), confirming the design rationale.

## Introduction

Dementia has grown as a major health and societal challenge nowadays, and its impact will be even more profound as the global population continues to age. The number of patients affected by dementia—with Alzheimer’s disease (AD) being the most frequent type—will increase from 35 million to an astonishing 135 million by 2050.^1^ Already 60% of them live in low- and middle-income countries, but by 2050, this will rise to 71%.^1^

The current lack of a cure is magnifying the problems of AD. While drugs (three acetylcholinesterase inhibitors (AChEI) and memantine) exist, they can only alleviate symptoms of dementia but are not able to halt the progression of the degenerative process. Often referred to as the “valley of death”, there is a large gap between basic research and translation to novel therapeutics. More than 200 drug candidates have failed in late-stage clinical trials, with a success rate of 0.4%, as compared with about 20% for cancer drugs.^2^ Over more than 400 clinical trials, there has been only one novel agent approved for AD since 2003 (GV-971 was approved in China in 2019 and is available only in China).^3^

As cases increase in populous countries like India, Brazil, and Indonesia, dementia will be an even more complex problem, especially in terms of an equitable access to treatments. Thus, there is a need to develop new medicines that are not only effective but also accessible, with no financial constraint.

The multifactorial nature of AD has been called into question as one of the factors contributing to the current lack of an effective drug therapy. The complex biology of AD is difficult to reduce to a single target whose modulation will impact the broad spectrum of pathologies and symptoms.^4^ More likely, AD is thought to be caused by a systemic breakdown of brain physiological networks.^5,6^ These have evolved to be very robust and redundant so that they are relatively insensitive to perturbations, with modulation by currently available single-target drugs having only a small, temporary effect. Conversely, treatments directed to multiple targets of the network would appear to have more chance of success.^5,7,8^

In 2008, we were among the first to propose single-molecule polypharmacology we named “multi-target-directed ligands” (MTDLs), as opposed to the available single-target drugs.^9^ Still today, these multifunctional molecules are considered a valuable option to effectively treat AD^10−12^ and similarly complex neurodegenerative diseases.^10^

In the years, we have realized that we are called not only to create drugs more adequate to face AD complexity but also to do so in a sustainable fashion so that the tools we develop are not only benign for the environment but also affordable and accessible to all the people and health systems that need them.

With this motivation, we have recently explored the possibility of developing new pharmacological tools for AD starting from cashew nutshell liquid (CNSL), an inexpensive and inedible food waste.^13,14^

Herein, we report the first sustainable MTDLs derived from CNSL, obtained by applying a rational framework combination approach.

## Results and Discussion

### Design

The concept of sustainability in its different nuances has been percolating the pharma’s activities for the past two decades.^15^ It has been embodied especially in the use of nontoxic solvents, biocatalytic processes, and waste minimization. Another still underexplored, yet increasingly important opportunity, is the use of biomass and waste feedstocks as a starting material for the development of new biologically active compounds and drugs.^16^ According to the #7 principle of Green Chemistry, using renewable resources, like microbial or plant biomass, offers a real alternative to traditional petrochemical intermediates.^16^

In addition to the clear environmental advantages, we believe that once properly optimized, the production of drugs from an inexpensive waste material may generate more affordable medicines and contribute to the goal of achieving Universal Health Coverage.^17^

With these concepts in mind, we undertook the task of developing an MTDL for AD starting from CNSL. Although we have already developed acetylcholinesterase (AChE)^13^ and histone deacetylase (HDAC)^14^ inhibitors with therapeutic potential for AD, this is the first time that a CNSL-derived MTDL has been rationally designed. To do so, we exploited a framework combination approach,^8^ which is the gold standard for creating new MTDLs, starting from compounds (or their pharmacophores) with the desired activity toward two targets of interest. A plethora of such hybrid molecules has been developed (for a recent review of the field, see the book by Decker^18^). The conjugation of tacrine with a second pharmacophoric moiety, pioneered by Pang et al.,^19^ is still an area of active research and development.^12,20^ As a key element of originality, this is the first report where one of the parent frameworks is derived from biomass.

**Figure 1:**
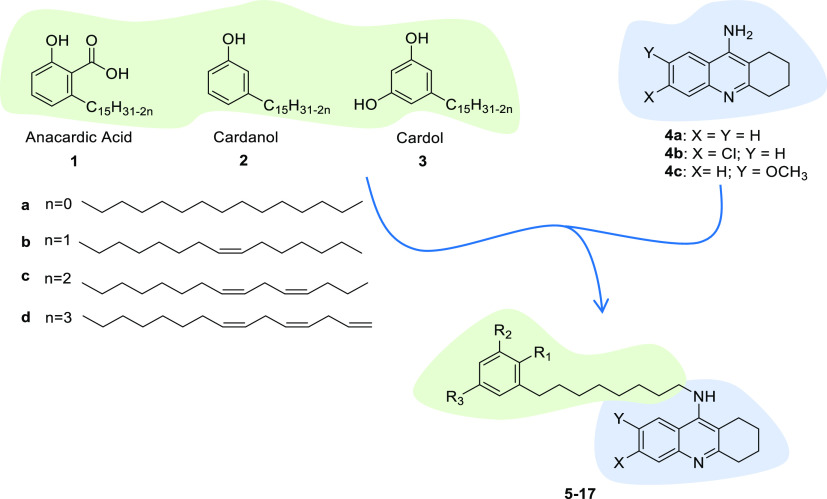
Design strategy toward hybrids **5**–**17** (see Table 1 for individual structures), starting from CNSL constituents **1**–**3** and tacrine templates **4a**–**4c**.

Our starting point was the remarkable anti-inflammatory activity showed by anacardic acid^21^ (**1**, Figure 1), a major constituent of CNSL. CNSL is an abundant byproduct derived from cashew (*Anacardium occidentale* L.) processing as well as a renewable source of long-chain phenols, i.e., anacardic acids (**1**), cardanols (**2**), and cardols (**3**), each present as a mixture of (un)saturated enomers. In traditional folk medicine, CNSL has also been reported to have several medicinal properties, including anti-inflammatory, analgesic, and antitumoral effects, in addition to indications as a larvicide.^22^ We were particularly intrigued by the anti-inflammatory properties of **1a**.^23^ These properties were recently found even superior to those of the anti-inflammatory drugs acetylsalicylic acid and dexamethasone in an in vitro cellular model.^23^ Particularly, **1a** has been shown to exert an immunoprotective effect by decreasing the expression of inflammatory genes.^23^

Mounting evidence indicates that inflammation has a causal role in AD pathogenesis, which is not limited to the neuronal compartment but involves strong interactions with immunological mechanisms in the brain.^24^ Accordingly, neuroinflammation is an appealing target for therapeutic intervention in AD.^25^

Thus, we reasoned that a hybrid of the well-established tacrine and tacrine analogues **4a**–**4c** (Figure 1) with that of **1**–**3** may furnish new MTDLs combining cholinesterase inhibition and antineuroinflammatory activity in a single molecule. The notion that the cholinergic system acts as an anti-inflammatory brake and that anticholinesterase drugs may positively modulate this process^26^ underlines a cross-talk between the two pathways and further supports our rationale. In fact, for maximizing efficacy, an MTDL should be directed to networked targets with established connectivity.^27^

As anticipated, **4a**–**4c** represent particularly effective frameworks for the design of MTDLs for AD.^28−30^ This is for several reasons: (i) tacrine is a drug with micromolar activity toward both AChE and butyrylcholinesterase (BChE); (ii) this dual inhibition is positive considering the importance of both cholinesterases in AD pathology;^31^ (iii) its high ligand efficacy allows combination with a second framework without exceedingly increasing molecular weight;^32^ and (iv) the potential hepatotoxicity linked to the tacrine scaffold could be overcome by conjugation with a second framework.^29^

As a peculiar consideration, it should be noted that tacrine and derivatives are easily synthesizable through environment-friendly and economical approaches (*e.g.*, catalysis with cheap and nontoxic catalysts and solvent-free reactions).^33^

Considering that the long alkyl chain (C15) of **1**–**3** might impair drug-like properties of the final compounds because of an excessive lipophilicity and an increase in molecular weight, our initial efforts focused on shorter (C8)-chain derivatives. In addition, we have assessed preliminary physicochemical properties and predicted blood–brain barrier (BBB) permeation of **1a** in a parallel artificial membrane permeability assay (PAMPA). The experimentally found p*K*_a_ value of 4.2 is the borderline with respect to the estimated p*K*_a_ limits for CNS penetration (between 4 and 10).^34^ Furthermore, we could not determine log *P* and effective permeability coefficient (*P*_e_) because of solubility problems (Table S1). On this basis, we decided to mainly focus on methylated derivatives (esters and ethers) and prepared the series of hybrids **5**–**17** (Figure 1).

### Chemistry

Compounds **5**–**17** were synthesized following the convergent approach depicted in Scheme 2, using functionalized CNSL C8 components (**21a**–**21d**) and tacrine analogues (**4a**–**4c**) as starting reagents. The synthetic route was developed with an eye on green chemistry principles (microwave (MW)-assisted, less hazardous or solvent-free reactions, use of continuous-flow reactors) to improve the overall process sustainability.

**Scheme 1:**
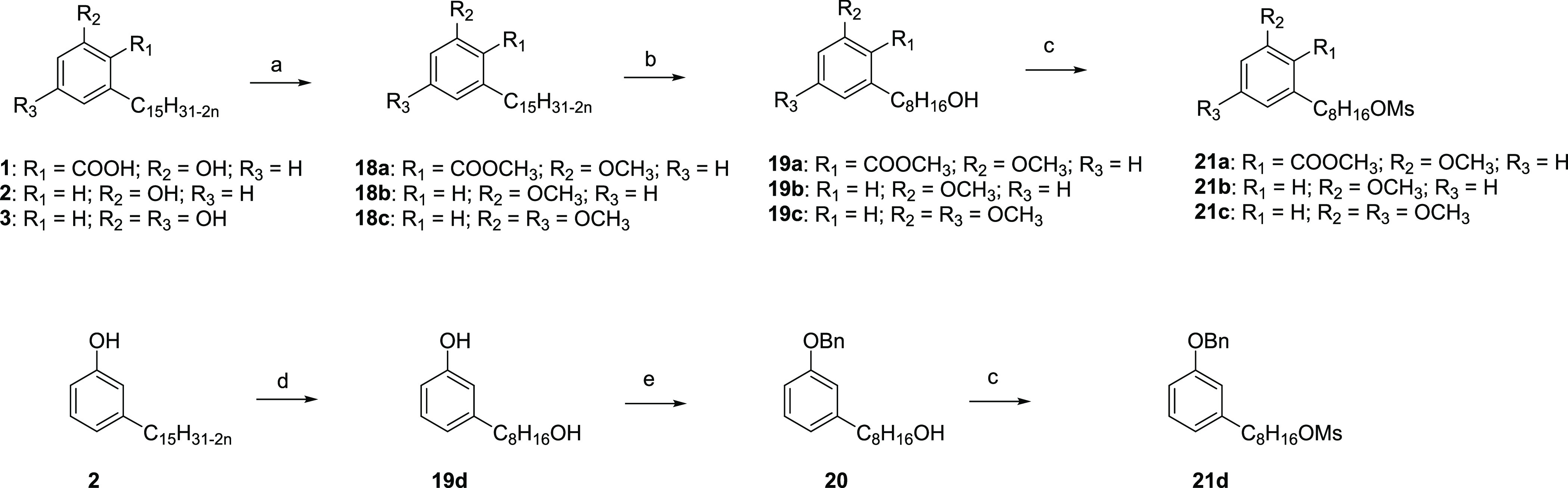
Synthesis of CNSL Mesylate Intermediates **21a**–**21d** Reagents and conditions: (a) K_2_CO_3_ and acetone; MeI, 24 h, and 110 °C (66–80%); (b) O_3_, DCM/MeOH, and 0 °C; NaBH_4_, 24 h, and rt (60–70%); (c) methanesulfonyl chloride, TEA, DCM, 12 h, and rt (60–85%); (d) acetic anhydride, MW: 450 W, and 3 min; O_3_, DCM/MeOH, and 0 °C; NaBH_4_, 16 h, and rt; HCl conc. (67%); and (e) benzyl bromide, K_2_CO_3_, acetone, 12 h, and reflux (92%).

**Scheme 2:**
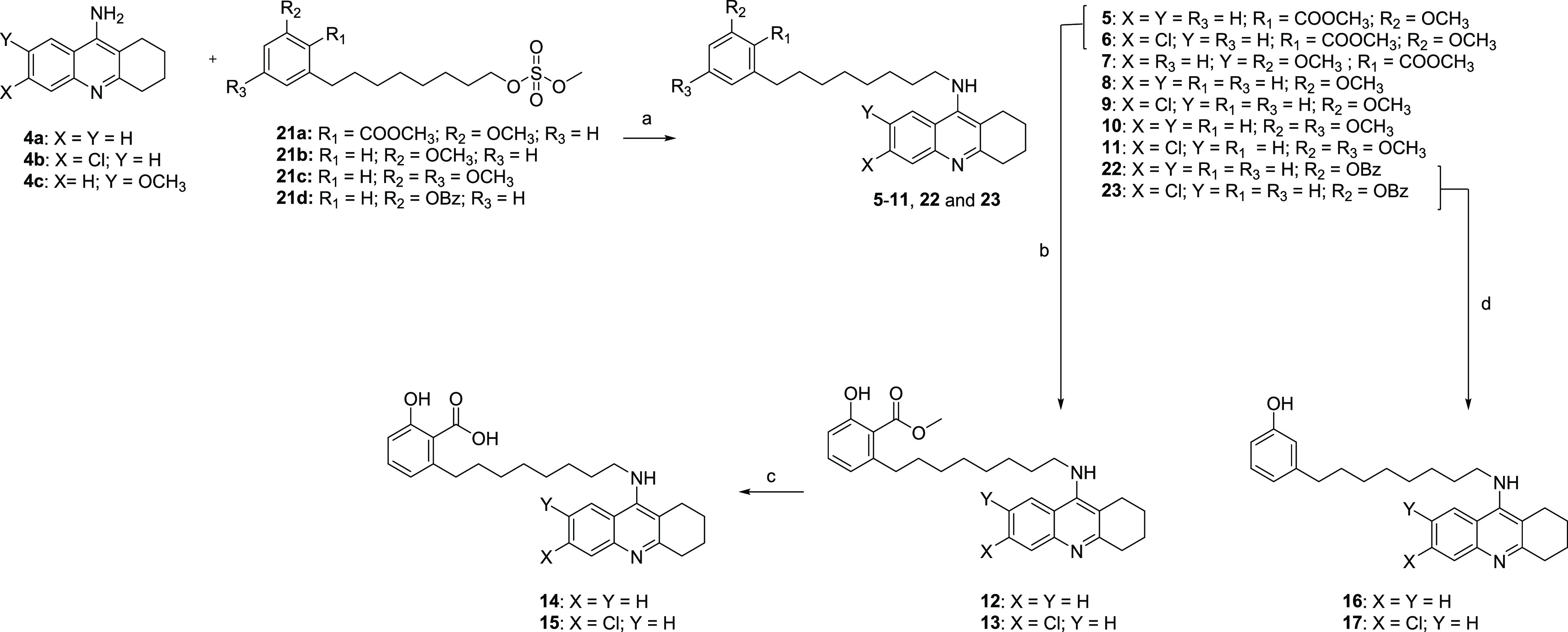
Synthesis of Final Compounds **5**–**17** Reagents and conditions: (a) KOH, DMSO, MW: 120 °C, and 12 min (20–34%); (b) BBr_3_, 0 °C to rt, DCM, and 40 min (25%); (c) KOH, MeOH/H_2_O, MW: 100 °C, and 10 min (54–84%); and (d) H-Cube H_2_, 5 bar, and Pd/C 10% (41–45%).

A mixture of the unsaturated natural components **1**–**3** was extracted from technical CNSL and isolated in good yield. After the extraction, **1**–**3** were methylated on acid and phenolic groups to obtain compounds **18a**–**18c**, respectively. Next, intermediates **18a**–**18c** underwent oxidative cleavage by ozonolysis and reduction with sodium borohydride of the resulting secondary ozonides to the corresponding alcohols, to give the C8 derivatives **19a**–**19c**, respectively (Scheme 1). To investigate the role of free phenolic and benzoic acid functions, cardanol **2** was transformed into its corresponding C8 primary alcohol, without undergoing the methylation step. Thus, before the ozonolysis reaction, **2** was acetylated with acetic anhydride to give a mixture of intermediates, which, after deprotection with HCl, provided **19d** in good yield. To differentiate the reactivity between the phenolic and aliphatic alcohol groups in **19d**, the phenol was protected as its benzyl ether **20**. The functionalized mesylate intermediates **21a**–**21d** were obtained by the reaction with methanesulfonyl chloride starting from **19a**–**19c** and **20**. In parallel, tacrine derivatives **4a**–**4c** were synthesized following the reported one-pot and solvent-free reaction of cyclohexanone with 2-aminobenzonitrile under zinc chloride catalysis.^35^ With the functionalized CNSL compounds in hand, the convergent synthesis based on an aliphatic substitution between **21a**–**21d** and tacrine derivatives **4a**–**4c** (Scheme 2) was applied. To synthesize derivatives **5**–**11**, **22**, and **23**, we developed an MW-assisted nucleophilic substitution procedure, which led to increases in yields and shortening of reaction time (from 12 h to 12 min), compared to a conventional heating protocol. Despite its utility in a nucleophilic substitution reaction, DMF is clearly not compatible with the drive toward more sustainable and environment-friendly medicinal chemistry development. Therefore, we opted for the safer DMSO.^36^ To investigate the role of free phenolic and benzoic acid functions, ether derivatives **5** and **6** were demethylated by using BBr_3_. Then, the methyl esters of **12** and **13** were hydrolyzed with KOH in MeOH/H_2_O under MW irradiation, to give **14** and **15**, respectively. The benzylic intermediates **22** and **23** were deprotected using Pd/C 10% as the catalyst in a continuous-flow hydrogenation reactor, to obtain the free phenolic compounds **16** and **17**, respectively. To note, continuous-flow processing has proved numerous advantages in terms of sustainability (cost, equipment size, energy consumption, waste generation, safety, and efficiency) over a traditional batch strategy.^37^

### Biology

To investigate the multitarget profile of the newly synthesized hybrids, they were screened for their cholinesterase and neuroinflammatory activities at enzymatic and cellular levels, respectively.

### hAChE and hBChE Inhibition Assays

To verify whether hybrids **5**–**17** could retain the anticholinesterase activity of the parent compounds **4a**–**4c**, we evaluated their inhibitory potency against human recombinant AChE (hAChE) and BChE from human serum (hBChE) by using the method of Ellman et al.^38^ IC_50_ values for all compounds were calculated and are reported in Table 1 in comparison with reference compounds **4a**–**4c**. In agreement with the literature data, we found that unsubstituted tacrine **4a** shows a slight selectivity toward hBChE, **4b** is a more potent hAChE inhibitor thanks to the positive effect of the chlorine atom,^39^ while the presence of a methoxy substituent (**4c**) is detrimental for both AChE and BChE inhibitions, albeit potentially positive in terms of metabolism/toxicity.^40^ Remarkably, all hybrids **5**–**17** showed an improved activity with respect to the parent compounds **4a**–**4c** (hAChE: 1.2- to 13.5-fold for tacrine derivatives and from 1.1- to 5.7-fold for Cl-tacrine derivatives; hBChE: 1.5- to 1300-fold for tacrine derivatives and from 4.2- to 1900-fold for Cl-tacrine derivatives), supporting the effectiveness of the framework combination design strategy. However, the AChE/BChE inhibitory profiles exhibit varying trends; thus, the SARs will be discussed separately for the two enzymes.

**Table 1:**
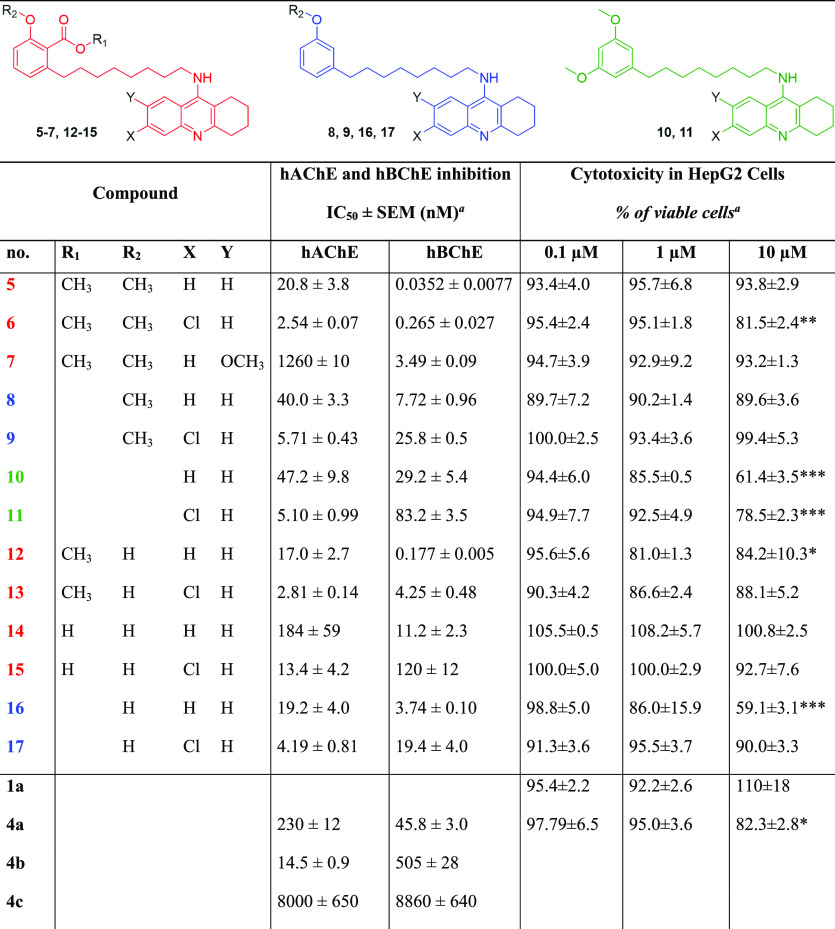
Cytotoxicity in HepG2 Cells and AChE and BChE Activities by **5**–**17** and Reference Compounds **4a**–**4c** and **1a**^b^

a Results are expressed as the mean of at least three experiments.

b *Significance was determined by ANOVA; Dunnett’s multiple comparison test *p* ≤ 0.05, **p ≤ 0.01, ***p ≤ 0.001, and ****p ≤ 0.0001.

All the hybrids were effective AChE inhibitors with potencies spanning two orders of magnitude (from nanomolar to single-digit micromolar) and with activity trends highly depending on the appended tacrine moiety. The most potent inhibitor was the dimethylated anacardic acid/Cl-tacrine hybrid **6** (IC_50_ = 2.54 nM), whereas the less active one was the dimethylated anacardic acid derivative carrying an OMe-tacrine unit **7** (IC_50_ equals to 1260 nM). This reinforces the finding that a Cl-tacrine moiety is more effective than an OMe-tacrine in recognizing the catalytic active site of the enzyme. As a confirmation of the tacrine scaffold as a driving force for AChE binding, within homogeneous subsets of hybrids, potency ranks in the following order: Cl-tacrine > tacrine > OMe-tacrine derivatives. As for the CNSL framework, methoxy-cardanols **8** and **9** and methoxy-cardols **10** and **11** exhibited similar profiles, suggesting that a second methoxy function on the aromatic ring does not provide additional interactions. Methylation of the anacardic acid salicylic moiety (both carboxylic and phenolic functions) was beneficial for affinity as dimethylated derivatives **5** and **6** and methyl esters **12** and **13** were more effective inhibitors than free anacardic acids **14** and **15**.

However, the most striking results were observed for hBChE inhibition, with three hybrids (**5**, **6**, and **12**) exhibiting subnanomolar potencies. Particularly, compared to AChE, an inverted rank order of potency was noted, with tacrine derivatives being more potent than Cl-tacrine counterparts, paralleling the selectivity trend of the parent compounds tacrine (**4a**) versus Cl-tacrine (**4b**). Indeed, this result is consistent with previous finding, showing that the insertion of a chlorine atom in the position 6 of the tacrine nucleus is detrimental for BChE inhibition because of steric hindrance.^39^ The presence of the anacardic acid framework seems important for enhancing the interaction with the enzyme. In fact, the anacardic acid subset of hybrids (**5**, **6**, and **12**–**15**) was more potent than the corresponding cardanol (**8**, **9**, **16**, and **17**) and cardol ones (**10** and **11**). Again, methylation of the anacardic acid at both the carboxylic and phenolic functions does make a favorable contribution to the activity as dimethylated **5** and **6** and methyl ester **12** were more potent than the free acid derivatives **14** and **15**. Particularly, **5**, **6**, and **12** were the top-ranked inhibitors of the series. Their exceptionally high activity toward hBChE (**5**, IC_50_ = 0.0352 nM; **6**, IC_50_ = 0.265 nM; and **12**, IC_50_ = 0.177 nM) is not completely unexpected as similar tacrine heterodimers carrying trimethoxy-substituted benzene units were found to be very potent inhibitors of BChE.^41^

BChE has traditionally been considered a surrogate for AChE in cholinergic neurotransmission; however, an increasing number of studies point to a key, unique role for BChE in AD.^42^ Actually, AChE levels are decreased by ∼50% in AD brains, whereas BChE levels increase by as much as 900% during disease progression.^43^ BChE is also associated with peculiar AD biomarkers, including Aβ oligomers and plaques as well as neurofibrillary tangles.^44^ However, the mechanisms underlying BChE involvement in AD progression are not completely understood. Thus, the development of potent and selective BChE inhibitors would improve understanding of the role of BChE in the aetiology of AD and lead to a wider variety of treatment options. Finally, inhibition of BChE seems to be associated to less severe side effects.^44^

In this respect, our most potent inhibitor (**5**) compares favorably with BChE inhibitors showing high potency and selectivity over AChE,^45−48^ including some multifunctional inhibitors recently reported.^49−51^ The high potency and selectivity found for **5** made this hybrid of interest for further studies.

### Crystal Structure of hBChE in Complex with **5**

In order to rationalize at a molecular level the excellent inhibitory potency of **5**, we solved its crystal structure in complex with hBChE. Crystallization, data collection and processing, and general structural analysis are reported in the Supporting Information.

**Figure 2:**
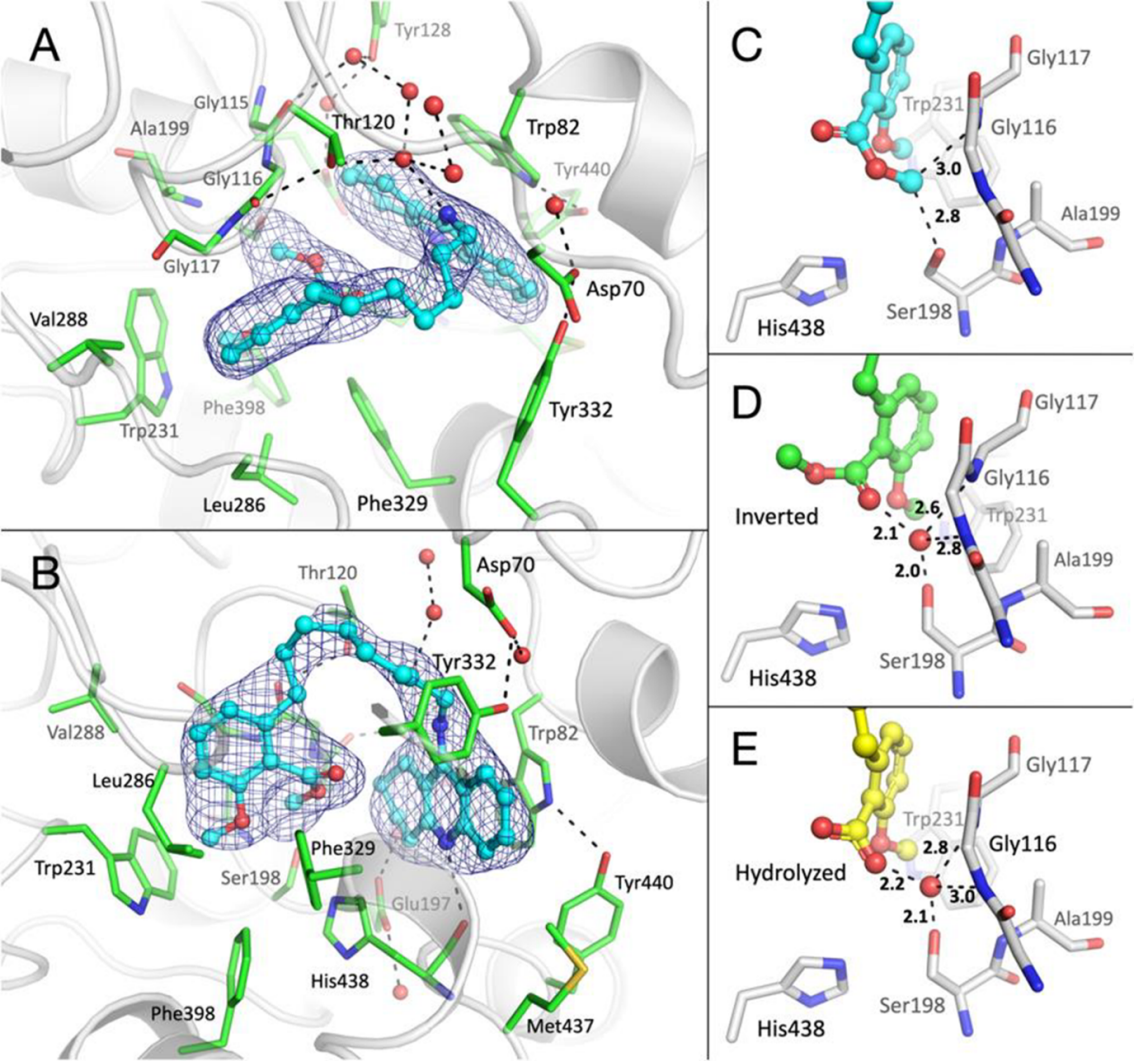
X-ray structure of **5** in complex with hBChE. Side view (A) and top view (B) of **5** in the active-site gorge of hBChE. The gorge is represented in white semitransparent cartoon with key residues as sticks and carbon atoms in green. The ligand is represented in ball and stick with carbon atoms in cyan. Oxygen atoms are represented in red, nitrogen atoms in blue, and sulfur atoms in yellow. Crystallographic water molecules are represented in red spheres and the dense hydrogen bond network in dashed lines. A 2.5 σ polder map^57^ calculated by omitting the ligand is represented as a blue mesh. Close-up view of the final model (C), inverted methyl carboxylate model (D), and hydrolyzed model (E) of the dimethyl anacardate moiety. Key interatomic distances are represented in dashed lines with values in Å.

Our structure revealed that **5** accommodates in the active-site gorge of hBChE without noticeable conformational adaptation of residues with respect to the unliganded parent structure (pdb entry: 1p0i). The tacrine moiety of **5** is in the same position as tacrine in the hBChE–tacrine complex (pdb entry: 4bds) with an rmsd of 0.26 Å.^52^ The key interactions are identical: the saturated cycle is embedded in the water molecule network, aromatic stacking with Trp82 (3.7 Å interplanar distance), hydrogen bonding between the pyridine nitrogen N26 and the main chain carbonyl of His438 (3.1 Å), and hydrogen bonding between the nitrogen atom N18 and a water molecule of the conserved water molecule network (2.9 Å) (Figure 2A,B). It is worth noting that the position of the tacrine moiety and its main stabilizing interactions are also identical in AChE in complex with alkylated tacrines or derivatives like huprines.^52,53^ Electron density for the C8 linker was not well defined in the first refinement cycles but is clearly visible in the polder map of the final structure when omitting the ligand (Figure 2A,B). This allows to constrain the orientation of the dimethyl anacardate moiety with the aromatic ring fitting into the groove of the acyl-binding pocket defined by residues Trp231, Leu286, Val288, Phe398, and Phe329, as commonly observed for other aromatic ligands,^54^ and the methoxy group pointing toward Trp231 (3.2 Å plane to methyl distance). The orientation of the methyl carboxylate substituent was more difficult to identify. The large peak of positive electron density in the initial |Fo| – |Fc| map corresponding to the dimethyl anacardate moiety is extending into the oxyanion hole defined by main chain NH of residues Gly116, Gly117, and Ala199. This density was initially modeled as a water molecule because it is usual to find one at this spot, bridging the catalytic serine to oxyanion hole residues, as seen for example in the hAChE–galantamine complex (pdb: 4ey6).^55^ Thus, we modeled the methyl carboxylate with its carbonyl oxygen (atom O8) pointing toward the water molecule in the oxyanion hole (Figure 2D). However, the interatomic distances between the water molecule and Ser198Oγ or O8 appeared to be unrealistically low (2.0 and 2.1 Å, respectively). So, we discarded this model. Alternatively, we made the hypothesis that the methyl carboxylate had been hydrolyzed knowing that benzoate esters are potential substrates of hBChE.^56^ However, modeling the substituent in its hydrolyzed carboxylate form and the water molecule in the oxyanion hole led to similar unrealistic interatomic distances (Figure 2E). Modeling of the molecule covalently bond to Ser198Oγ, i.e., benzoylated catalytic serine, or without a water molecule in the oxyanion hole also gave very poor unrealistic models.

Finally, modeling the methyl carboxylate with the methyl group in the oxyanion hole led to the most realistic structure (Figure 2C). In this model, O6 of the methyl ester is at close distance from C24 of the saturated ring (3.4 Å), and C7 is at the C–H hydrogen bond distance from Ser198Oγ (2.8 Å). Ser198 is an outlier in the Ramachandran plot (phi = 30° and psi = −100°), thus in an unfavorable conformation. Such an energy strained conformation has already been reported for this catalytic residue.^58^ Despite our efforts, a 6 σ positive peak remains unexplained in the |Fo| – |Fc| map, located in between Ser198Oγ, C7 of the methyl ester group, Gly116N, and Gly117N, suggesting that the final model is not perfect. The structure of the hBChE–**5** complex confirmed that tacrine is perfectly adapted to bind to the choline-binding pocket of hBChE. Disorder of the eight-carbon linker seen in the electron density suggests that it could be optimized by reducing its length. A five-carbon linker seems a good compromise between length and flexibility and could inspire further medicinal chemistry. The dimethyl anacardate subpart has a complementary shape to the acyl-binding pocket and oxyanion hole, which allow for optimized van der Waals interactions.

Altogether, the multiple interactions of the two aromatic moieties with the two main pockets of the active-site gorge completely enlighten the high affinity of compound **5** for hBChE.

### Cell-Based Screening

In parallel, a screening cascade with a set of assays was developed to identify those hybrids effectively modulating neuroinflammation at a cellular level. As the first step, the cytotoxicity profiles on the human liver cancer HepG2 (Table 1), human neuron-like SH-SY5Y (Figure S1), and murine microglial BV-2 (Figure S2) cell lines were determined. This was aimed to select those compounds endowed with an adequate safety–efficacy profile, a critical requirement for early-stage AD drug discovery.^59^ In fact, the failure of some AD candidates can be attributed to toxicity following drugs’ chronic administration.^59^ Particularly, in geriatric AD patients, due to aging, comorbidity, and subsequent polytherapy, there is an increased risk of drug–drug interactions and hepatotoxicity. Furthermore, tacrine (**4a**) has been withdrawn from the market for its severe side effects, the most notorious being hepatotoxic. Notwithstanding, this major clinical issue has been overcome in several cases and successful examples of tacrine hybrids with no hepatotoxicity have been reported.^60,61^ Thus, hepatotoxicity of **5**–**17** was preliminary assessed using a HepG2-based in vitro system, in comparison with parent compounds **1a** and **4a**. The HepG2 cell line has been extensively employed as a suitable in vitro system thanks to its homogeneous and consistent cellular features. We applied an experimental protocol we utilize in our laboratory, which shows 75–80% of residual cell viability for tacrine treatment (10 μM), following an incubation of 24 h.^62^ To further progress those molecules potentially devoid of hepatotoxicity, we set up an initial cutoff of >80% cell viability at 10 μM. Based on the collected data as well as on the cholinesterase inhibitory profiles (Table 1), compounds **5**, **6**, **9**, **12**–**15**, and **17** were selected as the most promising to continue the study. Neurotoxicity was evaluated by the MTT viability assay by treating human differentiated SH-SY5Y cells with increasing concentrations (0.1–1 μM) of the selected compounds for 24 h (Figure S1). Encouragingly, all the compounds were not significantly neurotoxic at both tested concentrations, being cell viability comparable or even slightly and significantly increased with respect to control cells.

The potential cytotoxicity of compounds **5**, **6**, **9**, **12**–**15**, and **17** was also evaluated on BV-2 microglial cells. BV-2 cells were treated with increasing concentrations (0.1–1 μM) of the selected compounds for 24 h, and cell viability was measured by the MTT assay (Figure S2). At the lowest concentration (0.1 μM), the compounds were not cytotoxic as cell viability of treated cells was comparable to the controls. At 1 μM, only compounds **5**, **6**, and **9** showed a slight, although significant, cytotoxicity.

### Antineuroinflammatory Activity

To assess the antineuroinflammatory potential, we subjected the less toxic compounds of our library (**5**, **6**, **9**, **12**–**15**, and **17**) to phenotype-based screening in murine microglial BV-2 cells. The discovery that patients with AD show increased levels of inflammatory mediators and the association among AD risk genes and the innate immune functions indicates a major role for neuroinflammation in AD pathogenesis.^63^ A key player in the neuroinflammatory response is microglia. Microglia are considered resident macrophages in the brain that provide a first line of defense in the central nervous system (CNS).^64^ Under physiological conditions, microglia play an important role in the development, structural formation, and functional regulation of the nervous system.^65−67^ After exogenous stimulation or microenvironment changes in the brain, resting microglia transform to an activated phenotype that leads to neuronal tissue damage and to the expression of neuroinflammation-related genes.^68,69^ Activated microglia release a large number of inflammatory factors, such as interleukin-1β (IL-1β) and tumor necrosis factor-α (TNF-α), by activating some signaling pathways, such as the Toll-like receptor 4 (TLR4), nuclear factor kappa B (NF-κB), and mitogen-activated protein kinase (MAPK) pathways.^70,71^ Moreover, activated microglia increase the expression of inducible nitric oxide synthase (iNOS)^68^ and COX-2.^72^ Therefore, inhibiting excessive microglial activation and reducing the production of proinflammatory mediators are potential avenues for controlling neuroinflammation in AD.

To study the anti-inflammatory activity of the selected compounds, BV-2 cells were exposed to bacterial endotoxin lipopolysaccharide (LPS) for 24 h. LPS is widely used as a proinflammatory agent as it induces inflammatory reactions both in primary microglia and BV-2 cells.^73,74^ The fact that 90% of the genes modulated by LPS in BV-2 cells are also induced in primary microglia and both show similar reaction patterns makes BV-2 cells a valid substitute for primary microglia in many experimental settings.^75^ Based on the cytotoxicity results (Figures S1 and S2) as well as the nanomolar cholinesterase potencies (Table 1), the experiments were carried out using concentrations in the 0.01–0.1 μM range.

**Figure 3:**
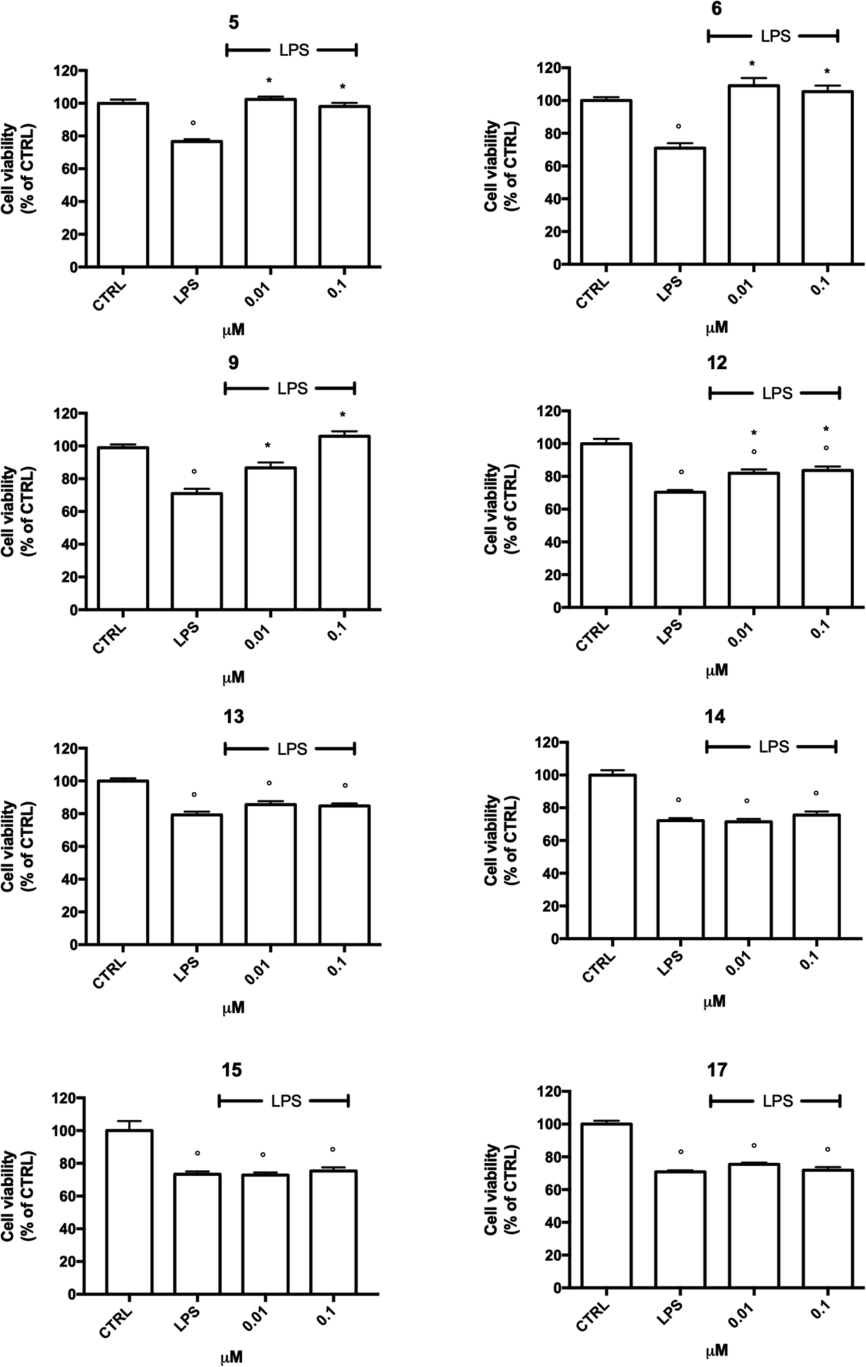
Anti-inflammatory effects of **5**, **6**, **9**, **12**–**15**, and **17** against LPS in BV-2 cells. BV-2 cells were treated with increasing concentration of the selected compounds (0.01–0.1 μM) for 24 h and exposed to 100 ng/mL LPS for further 24 h, and cell viability was evaluated by the MTT assay. Each bar represents means ± SEM of at least four independent experiments. Data were analyzed by one-way ANOVA followed by Tukey’s test. ^○^*p* < 0.05 compared to CTRL; *p < 0.05 compared to LPS.

Thus, BV-2 cells were treated with **5**, **6**, **9**, **12**–**15**, and **17** for 24 h and subsequently exposed to 100 ng/mL LPS for further 24 h (Figure 3). As assessed by the MTT assay, LPS treatment induced a significant reduction of cell viability. Anacardic acid (**13**–**15**) and cardanol (**17**) hybrids showed no ability to counteract LPS-induced damage (cell viability of treated cells was comparable to cells exposed to LPS).

On the other hand, dimethylated anacardic acids **5** and **6**, methyl-cardanol **9**, and anacardic acid methyl ester **12** significantly counteracted LPS-induced cell death. In particular, while **12** showed a moderate, although significant, effect already at 0.01 μM, **9** afforded total protection against LPS only at the highest tested concentration (0.1 μM). Remarkably, both dimethylated anacardic acid derivatives **5** and **6** (irrespective of the tacrine-appended moiety) restored cell viability to normal levels already at the very low concentration of 0.01 μM.

Of note, **5** and **6** were the most effective compounds not only in counteracting LPS-induced damage but also in inhibiting hBChE and hAChE, respectively. For this reason, their anti-inflammatory profile was investigated more in depth by evaluating their ability to modulate the expression of major neuroinflammatory cytokines, i.e., IL-1β, TNF-α, and mediators, i.e., iNOS and COX-2.

IL-1β and TNF-α are proinflammatory neurotoxic cytokines that contribute to neuronal dysfunction and neuronal loss in AD.^76^ iNOS is responsible of the formation of NO, one of the main cytotoxic mediators participating in the innate immune response in mammals. iNOS is not usually expressed in the brain. However, activated microglia are a major cellular source of iNOS. The excessive release of NO by activated microglia correlates with the progression of neurodegenerative disorders. Similarly, COX-2 has been associated with neurotoxicity, and inhibition of COX-2 induction reduces brain injury and delays the progress of neurodegenerative diseases.^77^

**Figure 4:**
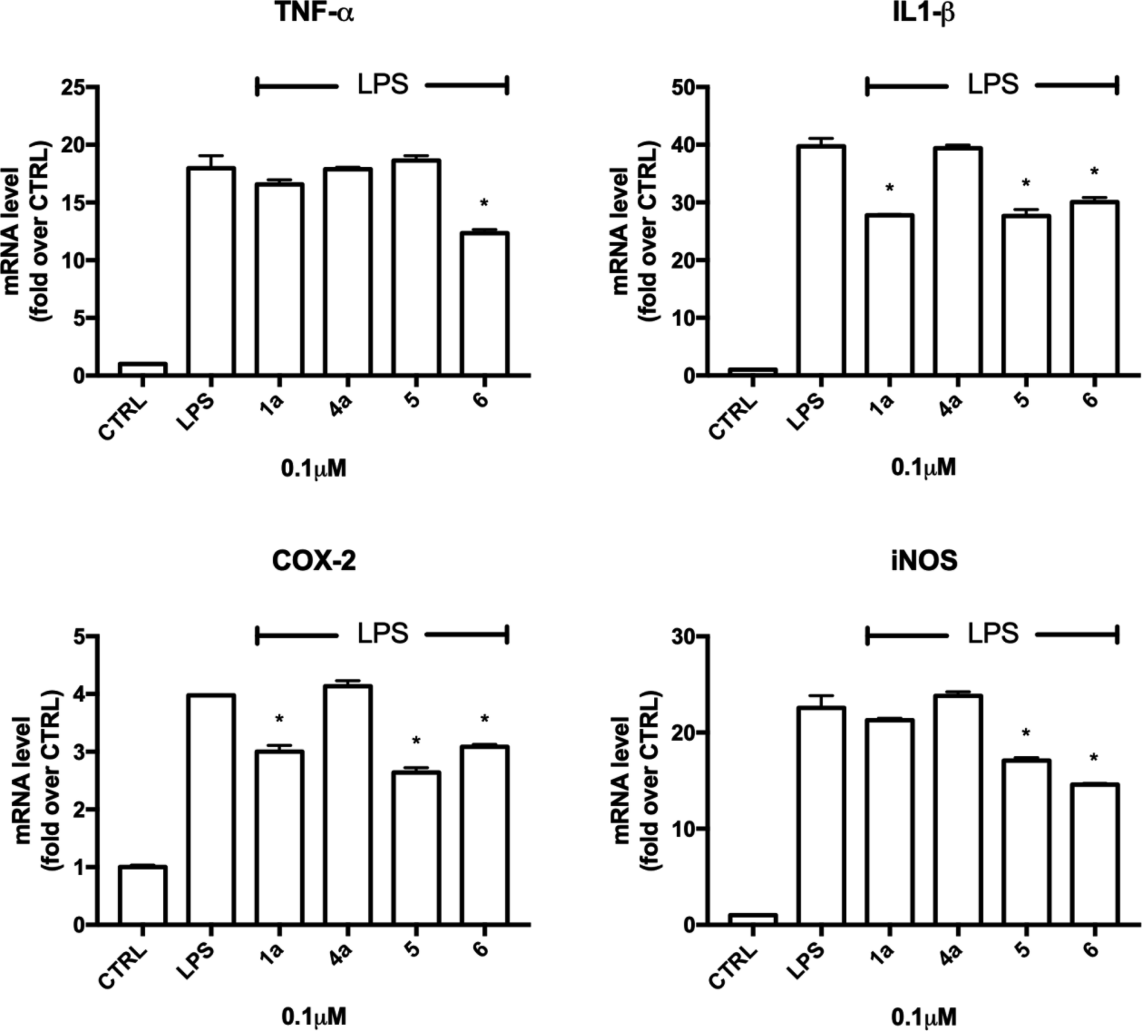
Expression of proinflammatory cytokines and enzymes in LPS-induced BV-2 cells treated with **1a**, **4a**, **5**, and **6**. BV-2 cells were treated with **1a**, **4a**, **5**, and **6** (0.1 μM) for 24 h and exposed to 100 ng/mL LPS for further 24 h, and real-time PCR was performed to detect TNF-α, IL-1β, iNOS, and COX-2 mRNA levels. Data are expressed as relative abundance vs CTRL. Each bar represents means ± SEM of three independent experiments. Data were analyzed with a one-way ANOVA followed by Tukey’s test. *p < 0.05 compared to LPS.

BV-2 cells were treated with **5** and **6** for 24 h and exposed to LPS 100 ng/mL for further 24 h, and the expression of the inflammatory mediators was evaluated by real-time polymerase chain reaction (RT-PCR) (Figure 4). Parent compounds **1a** and **4a** were added in the experimental setting as positive and negative controls, respectively. Intriguingly, parent compound **1a** chemically resembles salicylic acid. Interestingly, de Souza et al. demonstrated that it has higher anti-inflammatory activity than acetylsalicylic acid in vitro.^23^ Based on these considerations, we chose **1a** instead of salicylic/acetylsalicilic acids as a positive control. LPS significantly increased the expression of TNF-α, IL-1β, COX-2, and iNOS compared to control cells. As expected, **4a** was not effective, while **1a**, **5**, and **6** efficiently suppressed the transcription of COX-2 and iNOS genes and proinflammatory cytokines. Of note, both **5** and **6** showed a higher anti-inflammatory activity than **1a**. In fact, **6** was able to significantly reduce the expression of all the tested inflammatory mediators, **5** significantly downregulated IL-1β, COX-2, and iNOS (and not TNF-α), while **1a** reduced only the expression of IL-1β and COX-2. The higher anti-inflammatory activity of **5** and **6** compared to **1a** seems to support the effectiveness of the applied framework combination approach.

Notably, evidence indicates the cholinergic system as a mediator of neuroimmune interactions^26^ and BChE as an important player in regulating intrinsic inflammation and activity of cholinoceptive glial cells.^43^ Thus, it could be speculated that the higher activity of **5** and **6** compared to **1a** could be due to their concomitant modulation of both pathways. However, the possibility that it could be due to the different cell bioavailability of the two compounds cannot be ruled out.

Our results on **1a**’s anti-inflammatory activity are partially in agreement with the data of de Souza et al.,^23^ showing that **1a** (LDT11) is able to significantly reduce the expression of TNF-α, iNOS, COX-2, NF-κB, IL-1β, and IL-6. This discrepancy could be related to the different concentrations used (50 μM vs 0.1 μM). In addition, de Souza et al. investigated the anti-inflammatory activity of **1a** in a different model, i.e., the RAW264.7 murine macrophage cell line.^23^ As expected, **4a** did not influence the expression of all the tested inflammatory mediators.

**Figure 5:**
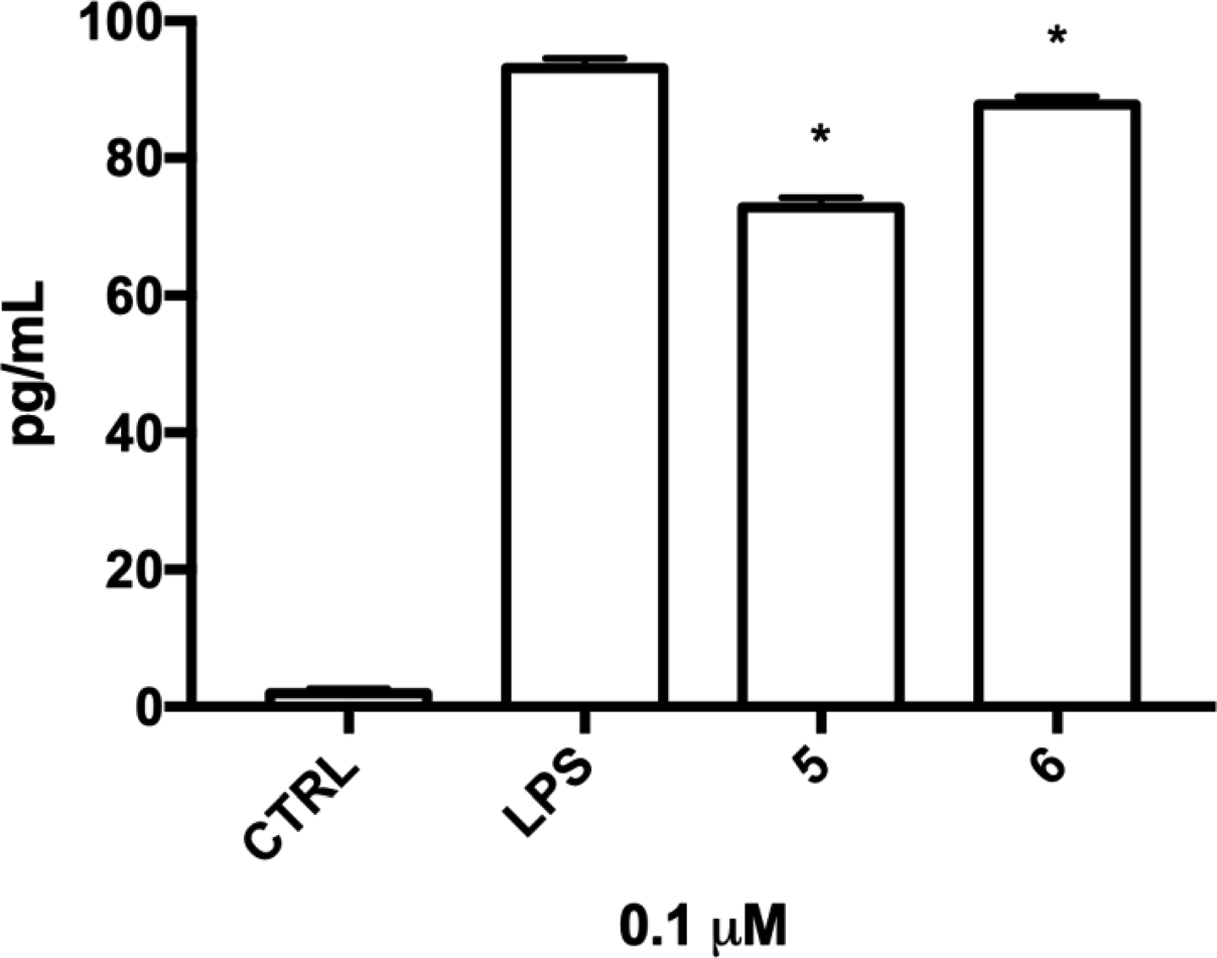
Secretion of IL1-β in activated BV-2 cells treated with **5** and **6**. BV-2 cells were treated with **5** and **6** (0.1 μM) for 24 h and exposed to 100 ng/mL LPS for further 24 h, and ELISA was performed to detect IL1-β concentration in the culture medium. Each bar represents means ± SEM of three independent experiments. Data were analyzed with a one-way ANOVA followed by Tukey’s test. *p < 0.05 compared to LPS.

The anti-inflammatory activity of **5** and **6** has been also confirmed by ELISA (Figure 5). The pretreatment with **5** and **6** was able to significantly decrease the release of IL-1β in the culture medium when compared to LPS-treated cells.

**Figure 6:**
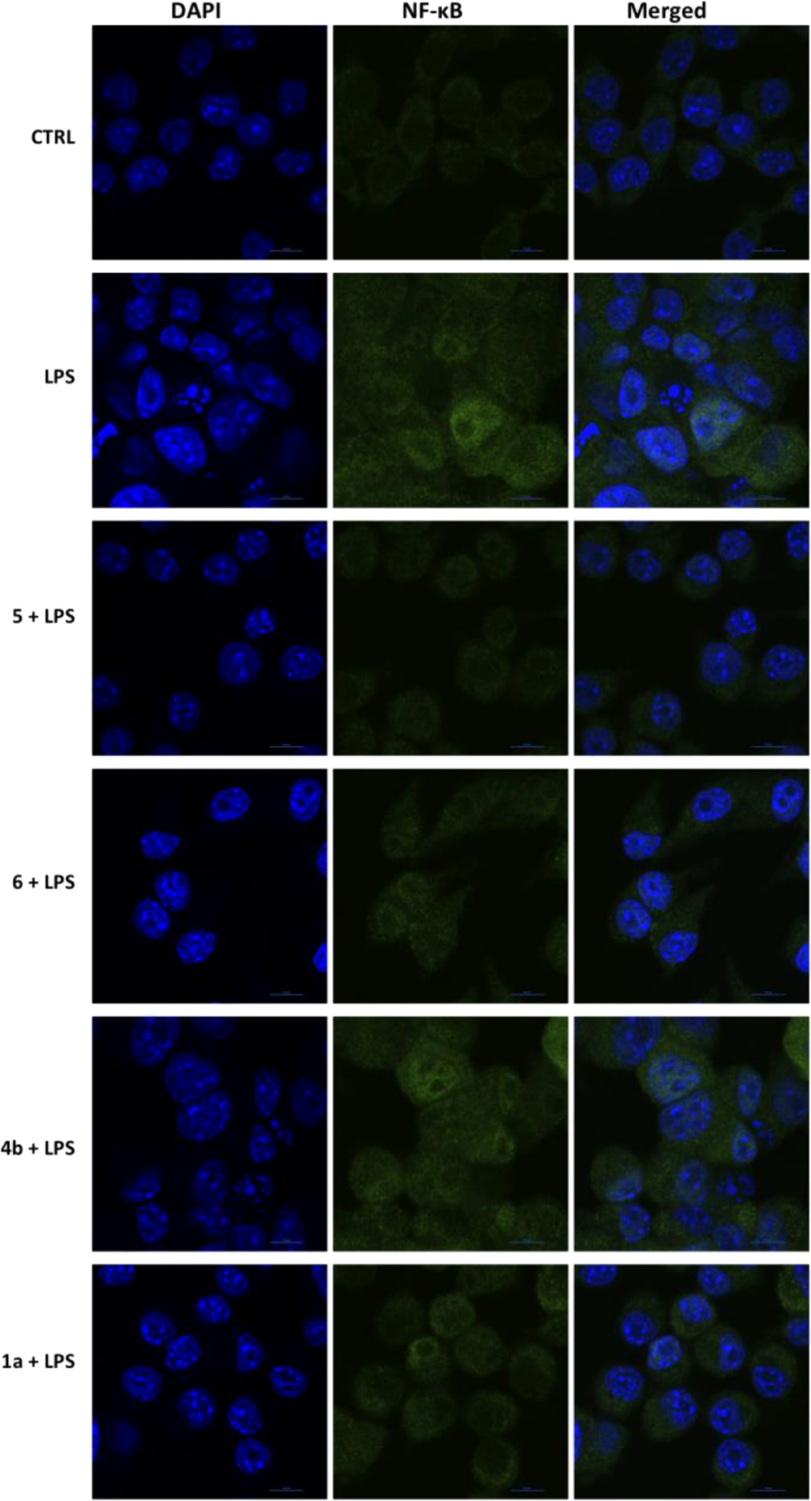
Nuclear translocation of NF-κB in LPS-induced BV-2 cells treated with **1a**, **4a**, **5**, and **6**. Cells were treated with compounds **5** and **6** (0.1 μM) and **1a** and **4a** as reference compounds (0.1 μM) for 24 h and then exposed to 100 ng/mL LPS for further 24 h. BV-2 cells were immunostained with a primary antibody against NF-κB p65 followed by secondary Alexa Fluor 488-conjugated antirabbit IgG antibody (green), and cell nuclei (blue) were visualized with DAPI. Scale bars: 10 μm.

The increased expression of proinflammatory enzymes and cytokines is mediated by the migration of transcription factor NF-κB to the nucleus. NF-κB is usually located in the cytoplasm in association with IκBα. Upon IκBα phosphorylation and degradation, NF-κB is isolated and translocated to the nucleus.^78,79^ Therefore, we further aimed to determine whether **5** and **6** could modulate LPS-induced nuclear translocation of NF-κB. BV-2 cells were treated with 0.1 μM **5** and **6** for 24 h and exposed to LPS for further 24 h, and the localization of the transcription factor NF-κB was evaluated by confocal immunofluorescence (Figure 6). **1a** and **4a** were used as reference compounds. LPS induced a strong increase in NF-κB levels both in the cytoplasm and in the nucleus. Consistent with the RT-PCR results, LPS-mediated nuclear translocation of NF-κB was considerably blocked by pretreatment with **5** and **6** and, at a lower extent, with **1a**. This suggests that **5** and **6** counteract neuroinflammation by inhibiting the transcriptional activation of NF-κB. This finding also confirms their higher neuroinflammatory activity with respect to **1a**.

### Blood–Brain Barrier Permeability Prediction

A key feature for AD drugs is their effective delivery into the brain at therapeutic concentrations, mainly because of the BBB presence. PAMPA-BBB is an in vitro tool developed to rapidly predict passive BBB permeation. BBB permeability of the most promising **5** and **6** was estimated using the PAMPA-BBB model in comparison with standard drugs, including AChEI tacrine (**4a**) and donepezil. The measurement predicted that both **5** and **6** have the potential to cross the BBB. Particularly, *P*_e_ values matched those of two standard AD drugs (donepezil and **4a**), known for effective BBB penetration (Table 2).

**Table 2:** In Vitro Permeability (*P*_e_) Values with Related Predictive Penetrations into the CNS of Commercial Drugs, **5** and **6**

	BBB penetration estimation	
compound	*P*_e_ ± SEM (× 10^–6^ cm/s)^a^	CNS (+/−)
**5**	6.99 ± 1.04	CNS +
**6**	17.70 ± 4.63	CNS +
furosemide	0.19 ± 0.07	CNS –
ranitidine	0.35 ± 0.31	CNS –
donepezil	21.93 ± 2.06	CNS –
tacrine (**4a**)	5.96 ± 0.59	CNS –

a *P*_e_ ± SEM (*n* = 3). Each compound was assessed in quadruplicate.

### Plasma Stability Assay

**Figure 7:**
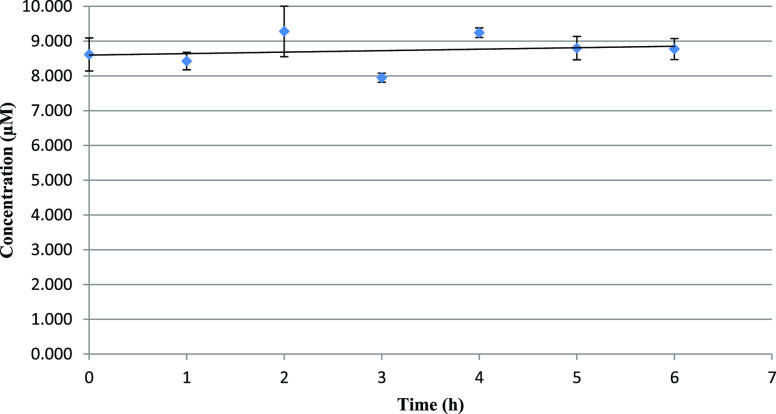
Human plasma stability of compound **5** upon incubation at 37 °C. Concentration was assessed by means of HPLC–MS. Analyses were performed in duplicate.

Considering that both **5** and **6** carry a labile ester functionality, we preliminary performed a human plasma stability assay. As determined by HPLC–MS analysis (Figure 7), **5** showed no decomposition over a 6 h range. This suggests that **5** is stable and not eventually transformed into the less active demethylated **14** in this time frame.

## Conclusions

After decades of massive research efforts and continuous clinical failures, the reason to target more than one pathway in AD seems rather clear. Inhibition of BChE is a promising target to increase the acetylcholine level and attenuate the cognitive decline, especially at a late disease stage. It could be also beneficial for modulating other AD hallmarks as a link between BChE and amyloid, and tau pathologies have been proposed. Furthermore, there is much evidence indicating that neuroinflammation and microglia are major contributors to AD. As such, molecules inhibiting proinflammatory microglia and neuronal death have been intensively investigated as possible disease-modifying drugs, although not exempt from complications.

In this study, we have developed a new series of hybrids by combining the cholinesterase activity of tacrine derivatives **4a**–**4c** with the anti-inflammatory properties of CNSL-derived **1**. Enzymatic studies have disclosed potent and selective inhibitors of both AChE and BChE. Compounds **5**, **6**, and **12**, endowed with subnanomolar activities, can be listed among the most effective BChE inhibitors so far developed. Specifically, to the best of our knowledge, **5** stands as the one with the lowest IC_50_ value (0.0352 nM). Our investigation in BV-2 microglial cells has revealed a protective activity against neurotoxic insults for **5** and **6** already at the remarkably low concentration of 0.01 μM. Both suppress LPS-induced IL-1β, COX-2, and iNOS (TNF-α only for **6**) overexpression. Particularly, they are capable of counteracting neuroinflammation by inhibiting the transcriptional activation of NF-κB, without causing cytotoxicity in microglial, neuronal, and hepatic cell lines.

On the basis of the obtained biological and PAMPA-BBB data, we can speculate that **5** and **6** may access the brain at their active nanomolar concentration.

Finally, as an important remark, the utilization of components from CNSL, a cheap and highly available food waste, offers an invaluable resource for developing new MTDLs for combating AD. It could be of importance and economic feasibility in low- and middle-income countries that are among the cashew top producers and, at the same time, areas with high current and future disease prevalence. In principle, the new molecules, in addition to the peculiar advantages of MTDLs, could offer accessible drugs to patients living in those countries, who might otherwise be excluded from access to therapy. Clearly, this is a long-term aspirational goal; however, in our opinion, it deserves attention by the medicinal chemistry community for the potential benefits to the global patient population and the environment.

## Experimental Section

### Chemistry

All the commercially available reagents and solvents were purchased from Sigma-Aldrich, Alfa Aesar, VWR, and TCI and used without further purification. Reactions were followed by analytical thin-layer chromatography (TLC) on precoated TLC plates (layer: 0.20 mm silica gel 60 with a fluorescent indicator UV254, from Sigma-Aldrich). Developed plates were air-dried and analyzed under a UV lamp (UV 254/365 nm). A CEM Discover SP focused microwave reactor was used for microwave-assisted reactions. Nuclear magnetic resonance (NMR) experiments were run on a Varian VXR 400 (400 MHz for ^1^H and 100 MHz for ^13^C). ^1^H and ^13^C NMR spectra were acquired at 300 K using deuterated chloroform (CDCl_3_) and methanol (CD_3_OD) as solvents. Chemical shifts (δ) are reported in parts per million (ppm) relative to tetramethylsilane (TMS) as the internal reference, and coupling constants (*J*) are reported in hertz (Hz). The spin multiplicities are reported as s (singlet), br s (broad singlet), d (doublet), t (triplet), q (quartet), and m (multiplet). Mass spectra were recorded on a Waters ZQ4000, XevoG2-XSQTof, Acquity arc-QDA LC–MS apparatus with electrospray ionization (ESI) in positive mode. Catalytic hydrogenation was performed on an H-Cube continuous-flow hydrogenation reactor (H-Cube, ThalesNano Nanotechnology, Budapest, Hungary). Compounds were named following IUPAC rules as applied by ChemBioDraw Ultra (version 16.0). The purity of compounds was determined using a Kinetex 5 μM EVO C18 100 Å, LC column 150 × 4.6 mm and an HPLC JASCO Corporation (Tokyo, Japan) instrument (PU-1585 UV equipped with a 20 μL loop valve). All the tested compounds (except compound **9**, whose purity is 94%) showed ≥95% purity by analytical HPLC.

#### Extraction of Anacardic Acid Mixtures (**1**) from Natural CNSL

A solution of 15 g of calcium hydroxide in methanol/water (6:1, 210 mL) was added to 30 g of natural CNSL. The system was stirred at 60 °C for 3 h. After this period, the mixture was concentrated under vacuum and filtered. The solid was transferred to a 1 L Erlenmeyer in which were added ethyl acetate (150 mL), distilled water (50 mL), and 50% HCl solution to reach pH = 1.0. The resulting solution was washed with saturated sodium chloride solution (50 mL) and dried over anhydrous Na_2_SO_4_. The solvent was evaporated under reduced pressure, and the mixture was purified by silica gel column chromatography (hexane/ethyl acetate: 0–30%), giving 16.5 g of the anacardic acid mixture (**1**), corresponding to approximately 55% of the mass of natural CNSL used.

#### Extraction of Mixtures of Cardanols (**2**) and Cardols (**3**) from Technical CNSL

Twenty grams of technical CNSL donated from the company Resibras was purified by silica gel column chromatography (hexane/ethyl acetate: 5–35%) to provide 14 g of the mixture of cardanols (**2**, 70% of the applied mass) and 4.8 g of the mixture of cardols (**3**, 24% of the applied mass).

#### General Procedure I (for Compounds **18a**–**18c**)

Mixtures of **1** (12.5 g, 36.5 mmol) or **2** (10 g, 33.5 mmol) or **3** (6 g, 18.7 mmol) were stirred with potassium carbonate (3.0 equiv) in acetone (300 mL). After 2 h, methyl iodide (4.0 or 6.0 equiv) was added, and the reaction was refluxed at 110 °C with a cooling system at −8 °C for 24 h. After cooling, the solvent was evaporated in vacuo. Distilled water (40 mL) was added, and the residue was extracted with dichloromethane (3 × 30 mL). The combined organic phases were washed with 10% HCl solution (10 mL) and saturated sodium chloride solution (10 mL), dried anhydrous Na_2_SO_4_, filtered, and concentrated under vacuum. The crude product was purified by column chromatography (hexane/ethyl acetate: 5–20%), providing the unchanged mixture of saturated derivatives and the mixtures of methyl *O*-methylanacardates (**18a**, 74%), *O*-methylcardanols (**18b**, 80%), and *O*,*O*-dimethylcardols (**18c**, 66%) with different unsaturation degrees as brown oils. The mixtures have been used for the further step without characterization.

#### General Procedure II (for Compounds **19a**–**19c**)

To an ozonation flask was added a solution (5.0 mmol, 1.0 equiv) of the O-methylated methylanacardate mixture **18a** or O-methylated cardanol **18b** or O,O-dimethylated cardol **18c** in dichloromethane/methanol (1:1, 60 mL). The reaction was cooled at 0 °C with an ice–water bath, and the solution was treated with ozone for approximately 3 h. After purging the reaction mixture with nitrogen, NaBH_4_ (20.0 mmol, 4.0 equiv) was added, and the mixture was left warming to room temperature, under vigorous stirring for 24 h. The reaction was quenched with water and 10% aqueous HCl (10 mL) and extracted with dichloromethane (3 × 30 mL). The combined organic layers were washed with brine solution (10 mL), dried over anhydrous Na_2_SO_4_, and evaporated. The residue was purified by chromatography (hexane/ethyl acetate: 20–35%), providing the corresponding alcohol.

#### General Procedure III (for Compounds **21a**–**21d**)

Compounds **19a**–**19c** or **20** (0.68 mmol, 1.0 equiv) and NEt_3_ (0.88 mmol, 1.3 equiv) were stirred in dichloromethane (0.35 mL) at 0 °C for 15 min. Methanesulfonyl chloride (0.88 mmol, 1.3 equiv) was added dropwise to the mixture and stirred overnight at room temperature. The reaction was quenched with water and extracted with dichloromethane (3 ×10 mL). The organic layers were collected, dried over anhydrous Na_2_SO_4_, filtered, and concentrated under vacuum. The crude products were purified by column chromatography.

#### General Procedure IV (for Compounds **5**–**11**, **22**, and **23**)

The appropriate compounds **4a**–**4c** (0.15 mmol, 1.0 equiv) were solubilized in dry DMSO (1.3 mL), together with KOH (0.24 mmol, 1.6 equiv) and 4 Å molecular sieves. The solution was stirred at room temperature for 1 h under N_2_. The derivatives **21a**–**21d** (0.18 mmol, 1.2 equiv) were then added to the mixture, and the reaction was carried out under microwave irradiation at 120 °C for 12 min. The mixture was poured into water and extracted with dichloromethane (3 × 10 mL). The organic layers were collected, dried over anhydrous Na_2_SO_4_, filtered, and concentrated under vacuum. The crude product was purified by column chromatography to afford the corresponding intermediate.

#### General Procedure V (for Compounds **12** and **13**)

BBr_3_ (1 mmol, 4.0 equiv) was added at 0 °C to a stirred solution of compound **5** or **6** (0.25 mmol, 1.0 equiv) in dichloromethane (3 mL). The resulting mixture was stirred at room temperature for 40 min. When completed, the reaction was quenched with a saturated solution of aqueous NaHCO_3_ and 20 mL of water. The mixture was extracted with dichloromethane (3 × 10 mL), and the organic layers were collected, dried over anhydrous Na_2_SO_4_, filtered, and evaporated to dryness under vacuum. The crude product was purified by column chromatography to afford the corresponding intermediate **12** or **13**.

#### General Procedure VI (for Compounds **14** and **15**)

Compound **12** or **13** (0.07 mmol, 1.0 equiv) was solubilized in 2 mL of 3.5 M KOH solution (water/methanol, 2:1), and the reaction was performed under microwave irradiation at 100 °C for 10 min. The mixture was cooled down at 0 °C, and a solution of 2 N HCl was added dropwise until pH = 2. After 20 min, the obtained white precipitate was collected by filtration. The filtrate was suspended in methanol, and the inorganic insoluble precipitate was filtered off. The organic phase was dried under vacuum to give the corresponding hydrochloride derivative **14** or **15**.

#### General Procedure VII (for Compounds **16** and **17**)

A ThalesNano H-Cube Mini flow reactor was equipped with a Pd/C 10% catalyst cartridge. The reactor was programmed to run at 25 °C and 5 bar. A solution of 0.01 M compounds **22** and **23** (0.07 mmol, 1.0 equiv) (ethyl acetate/methanol, 1:1) was pumped through the reactor at a flow rate of 1 mL/min. The crude product coming out of the exit port was collected, concentrated under vacuum, and purified by flash chromatography.

#### Methyl 2-Methoxy-6-(8-((1,2,3,4-tetrahydroacridin-9-yl)amino)octyl)benzoate (**5**)

The title compound was obtained according to general procedure IV using **4a** and **21a**. The crude product was purified by column chromatography (6.5:3:0.5 petroleum ether/ethyl acetate/NEt_3_). Compound **5** was obtained as a sticky yellow-brown oil. Yield: 20%. HPLC purity: 95%. ^1^H NMR (CDCl_3_, 400 MHz): δ 1.30–1.39 (m, 8H), 1.55–1.66 (m, 4H), 1.92 (m, 4H), 2.53 (t, 2H, *J* = 8.0 Hz), 2.70 (m, 2H), 3.06 (m, 2H), 3.47 (t, 2H, *J* = 7.6 Hz), 3.81 (s, 3H), 3.89 (s, 3H), 6.75 (d, 1H, *J* = 8.4 Hz), 6.80 (d, 1H, *J* = 7.2 Hz), 7.26 (t, 1H, *J* = 7.6 Hz), 7.33 (t, 1H, *J* = 7.2 Hz), 7.56 (t, 1H, *J* = 7.2 Hz), 7.90–7.96 (m, 2H). ^13^C NMR (CDCl_3_, 100 MHz): δ 22.3, 22.8, 24.5, 26.8, 29.1, 29.2, 29.2, 29.3, 30.9, 31.0, 31.6, 32.8, 31.7, 33.3, 33.4, 49.3, 51.9, 55.8, 108.4, 113.4, 121.4, 121.4, 122.9, 123.5, 123.8, 127.3, 128.9, 130.1, 141.1, 156.2, 168.8. HRMS (ESI+) *m*/*z*: [M + H]^+^ calcd for C_30_H_38_N_2_O_3_, 475.29552; found, 475.29597.

#### Methyl 2-(8-((6-Chloro-1,2,3,4-tetrahydroacridin-9-yl)amino)octyl)-6-methoxybenzoate (**6**)

The title compound was obtained according to general procedure IV using **4b** and **21a**. The crude product was purified by column chromatography (7.5:2:0.5 petroleum ether/ethyl acetate/NEt_3_). Compound **6** was obtained as a sticky yellow-brown oil. Yield: 34%. HPLC purity: 98%. ^1^H NMR (CDCl_3_, 400 MHz): δ 1.28–1.35 (m, 8H), 1.53–1.66 (m, 4H), 1.89 (s, 4H), 2.51 (t, 2H, *J* = 7.6 Hz), 2.63 (m, 2H), 3.04 (m, 2H), 3.50 (t, 2H, *J* = 7.2 Hz), 3.79 (s, 3H), 3.87 (s, 3H), 6.72–6.79 (m, 2H), 7.24 (m, 2H), 7.89–7.93 (m, 2H). ^13^C NMR (CDCl_3_, 100 MHz): δ 22.3, 22.7, 24.4, 26.7, 29.1, 29.1, 29.2, 30.9, 31.6, 33.1, 33.3, 49.4, 51.9, 55.8, 108.4, 115.0, 117.8, 121.4, 123.5, 124.4, 124.6, 126.5, 130.2, 141.1, 151.4, 156.3, 168.8. HRMS (ESI+) *m*/*z*: [M + H]^+^ calcd for C_30_H_37_ClN_2_O_3_, 509.256547; found, 509.25651.

#### Methyl 2-Methoxy-6-(8-((7-methoxy-1,2,3,4-tetrahydroacridin-9-yl)amino)octyl)benzoate (**7**)

The title compound was obtained according to general procedure IV using **4c** and **21a**. The crude product was purified by column chromatography (7.5:2:0.5 petroleum ether/ethyl acetate/NEt_3_). Compound **7** was obtained as a sticky yellow-brown oil. Yield: 23%. HPLC purity: 99%. ^1^H NMR (CDCl_3_, 400 MHz): δ 1.29–1.37 (m, 8H), 1.55–1.66 (m, 4H), 1.90 (m, 4H), 2.51 (t, 2H, *J* = 7.6 Hz), 2.69 (m, 2H), 3.05 (m, 2H), 3.42 (t, 2H, *J* = 6.8 Hz), 3.79 (s, 3H), 3.87 (s, 3H), 3.89 (s, 3H), 6.73–6.80 (m, 2H), 7.24 (m, 4H), 7.99 (d, 1H, *J* = 7.2 Hz). ^13^C NMR (CDCl_3_, 100 MHz): δ 20.9, 22.1, 24.2, 26.6, 28.8, 29.0, 29.1, 29.2, 30.9, 31.4, 33.3, 48.1, 52.1, 55.0 103.3, 108.4, 117.8, 121.4, 122.9, 123.2, 130.2, 141.0, 154.0, 156.2, 156.6, 168.3. MS (ESI+) *m*/*z*: [M + H]^+^ calcd for C_31_H_40_N_2_O_4_, 504; found, 505.

#### *N*-(8-(3-Methoxyphenyl)octyl)-1,2,3,4-tetrahydroacridin-9-amine (**8**)

The title compound was obtained according to general procedure IV using **4a** and **21b**. The crude product was purified by column chromatography (7:2.5:0.5 petroleum ether/ethyl acetate/NEt_3_). Compound **8** was obtained as a sticky yellow-brown oil. Yield: 24%. HPLC purity: 95%. ^1^H NMR (CDCl_3_, 400 MHz): δ 1.30–1.39 (m, 8H), 1.55–1.66 (m, 4H), 1.92 (m, 4H), 2.53 (t, 2H, *J* = 8.0 Hz), 2.70 (m, 2H), 3.06 (m, 2H), 3.47 (t, 2H, *J* = 7.6 Hz), 3.77 (s, 3H), 6.70–6.75 (m, 3H), 7.17 (t, 1H, *J* = 7.6 Hz), 7.35 (t, 1H, *J* = 7.2 Hz), 7.58 (t, 1H, *J* = 7.2 Hz), 7.99 (d, 1H, *J* = 8.8 Hz), 8.09 (d, 1H, *J* = 7.6 Hz). ^13^C NMR (CDCl_3_, 100 MHz): δ 21.5, 22.3, 22.6, 22.8, 24.0, 26.7, 29.1, 29.1, 29.3, 29.6, 31.2, 31.4, 35.9, 49.0, 55.0, 110.6, 114.2, 117.6, 120.8, 123.5, 124.4, 129.1, 130.6, 144.3, 153.4, 154.4, 159.5. MS (ESI+) *m*/*z*: [M + H]^+^ calcd for C_28_H_36_N_2_O, 416; found, 417.

#### 6-Chloro-*N*-(8-(3-methoxyphenyl)octyl)-1,2,3,4-tetrahydroacridin-9-amine (**9**)

The title compound was obtained according to general procedure IV using **4b** and **21b**. The crude product was purified by column chromatography (8:1.5:0.5 petroleum ether/ethyl acetate/NEt_3_). Compound **9** was obtained as a sticky yellow-brown oil. Yield: 29%. HPLC purity: 94%. ^1^H NMR (CDCl_3_, 400 MHz): δ 1.32–1.38 (m, 8H), 1.60–1.68 (m, 4H), 1.91 (m, 4H), 2.57 (t, 2H, *J* = 7.6 Hz), 2.65 (m, 2H), 3.05 (m, 2H), 3.51 (t, 2H, *J* = 7.6 Hz), 3.79 (s, 3H), 6.72–6.77 (m, 3H), 7.19 (t, 1H, *J* = 8.4 Hz), 7.27 (m, 1H), 7.90–7.93 (m, 2H). ^13^C NMR (CDCl_3_, 100 MHz): δ 22.4, 22.8, 24.4, 26.8, 29.1, 29.2, 29.3, 31.2, 31.7, 33.5, 35.9, 49.5, 55.1, 110.7, 114.2, 115.2, 118.0, 120.8, 124.3, 124.67, 126.9, 129.1, 134.3, 144.3, 151.1, 159.5. MS (ESI+) *m*/*z*: [M + H]^+^ calcd for C_28_H_35_ClN_2_O, 450; found, 451.

#### *N*-(8-(3,5-Dimethoxyphenyl)octyl)-1,2,3,4-tetrahydroacridin-9-amine (**10**)

The title compound was obtained according to general procedure IV using **4a** and **21c**. The crude product was purified by column chromatography (7.5:2:0.5:1:0.1 petroleum ether/ethyl acetate/dichloromethane/methanol/aqueous 32% ammonia). Compound **10** was obtained as a sticky yellow-brown oil. Yield: 25%. HPLC purity: 95%. ^1^H NMR (CDCl_3_, 400 MHz): δ 1.46–1.13 (m, 8H), 1.73–1.46 (m, 4H), 1.91 (t, 4H, *J* = 6.6 Hz), 2.58–2.39 (m, 2H), 2.68 (s, 2H), 3.08 (s, 2H), 3.49 (t, 2H, *J* = 7.1 Hz), 3.61 (t, 2H, *J* = 6.6 Hz), 3.75 (s, 6H), 6.46–5.79 (m, 2H), 6.76 (m, 1H), 7.28 (m, 1H), 7.54 (t, 1H), 7.95 (d, 2H, *J* = 8.6 Hz). ^13^C NMR (CDCl_3_, 100 MHz): δ 22.6, 24.4, 25.2, 26.8, 29.1, 29.3, 31.6, 33.4, 39.6, 49.9, 52.2, 54.8, 55.1, 57.6, 97.4, 106.5, 107.0, 108.3, 119.9, 121.4, 123.0, 123.8, 130.1, 141.0, 141.2, 145.1, 159.2, 160.6. MS (ESI+) *m*/*z*: [M + H]^+^ calcd for C_29_H_38_N_2_O_2_, 446; found, 447.

#### 6-Chloro-*N*-(8-(3,5-dimethoxyphenyl)octyl)-1,2,3,4-tetrahydroacridin-9-amine (**11**)

The title compound was obtained according to general procedure IV using **4b** and **21c**. The crude product was purified by column chromatography (7.5:2:0.5:1:0.1 petroleum ether/ethyl acetate/dichloromethane/methanol/aqueous 32% ammonia). Compound **11** was obtained as a sticky yellow-brown oil. Yield: 25%. HPLC purity: 95%. ^1^H NMR (CDCl_3_, 400 MHz): δ 1.40–1.10 (m, 8H), 1.57–1.62 (m, 4H), 1.91 (t, 4H, *J* = 2.9 Hz), 2.58–2.47 (m, 2H), 2.65 (s, 2H), 3.02 (s, 2H), 3.45 (s, 2H), 3.77 (s, 6H), 6.46–6.16 (m, 3H), 7.57–6.97 (m, 2H), 7.8–7.90 (m, 2H). ^13^C NMR (CDCl_3_, 100 MHz): δ 22.6, 22.8, 24.5, 26.8, 29.1, 29.2, 29.3, 31.1, 31.7, 33.9, 36.2, 49.5, 55.2, 97.4, 106.4, 115.5, 118.3, 124.1, 124.6, 127.4, 133.9, 145.1, 148.0, 150.8, 159.3, 160.6. MS (ESI+) *m*/*z*: [M + H]^+^ calcd for C_29_H_37_ClN_2_O_2_, 480; found, 481.

#### Methyl 2-Hydroxy-6-(8-((1,2,3,4-tetrahydroacridin-9-yl)amino)octyl)benzoate (**12**)

The title compound was obtained according to general procedure V starting from **5**. The crude product was purified by column chromatography (7:2.5:0.5:0.05 petroleum ether/dichloromethane/methanol/aqueous 32% ammonia). Compound **12** was obtained as a sticky colorless oil. Yield: 25%. HPLC purity: 99%. ^1^H NMR (CDCl_3_, 400 MHz): δ 1.25–1.38 (m, 8H), 1.50–1.52 (m, 2H), 1.64 (t, 2H, *J*_1_ = 8 Hz), 1.91 (s, 4H), 2.69 (s, 2H), 2.85 (t, 2H, *J*_1_ = 8 Hz), 3.06 (s, 2H), 3.48 (t, 2H, *J* = 7.1 Hz), 3.92 (s, 4H), 6.69 (d, 1H, *J* = 7.5 Hz), 6.83 (d, 1H, *J* = 8.2 Hz), 7.29 (dt, 2H, *J* = 15.8, 7.7 Hz), 7.54 (t, 1H, *J* = 7.5 Hz), 7.80–8.05 (m, 2H). ^13^C NMR (CDCl_3_, 100 MHz): δ 22.7, 23.0, 24.7, 26.9, 29.3, 29.3, 29.6, 31.7, 31.9, 33.8, 36.4, 49.4, 52.0, 112.1, 115.5, 115.6, 120.0, 122.2, 122.8, 123.5, 128.3, 128.4, 134.0, 145.8, 147.1, 150.9, 158.2, 162.2, 171.7. MS (ESI+) *m*/*z*: [M + H]^+^ calcd for C_29_H_36_N_2_O_3_, 460; found, 461.

#### Methyl 2-(8-((6-Chloro-1,2,3,4-tetrahydroacridin-9-yl)amino)octyl)-6-hydroxybenzoate (**13**)

The title compound was obtained according to general procedure V starting from **6**. The crude product was purified by column chromatography (7:2.5:0.5:0.05 petroleum ether/dichloromethane/methanol/aqueous 32% ammonia). Compound **13** was obtained as a sticky colorless oil. Yield: 25%. HPLC purity: 97%. ^1^H NMR (CDCl_3_, 400 MHz): δ 1.23–1.40 (m, 8H), 1.47–1.54 (m, 2H), 1.67 (dt, 2H, *J* = 8 Hz), 1.88–1.89 (m, 4H), 2.63 (s, 2H), 2.79–2.92 (m, 2H), 3.04 (s, 2H), 3.51 (t, 2H, *J* = 6.8 Hz), 3.92 (s, 3H), 4.19 (s, 1H), 6.69 (d, 1H, *J* = 7.5 Hz), 6.75–6.98 (m, 1H), 7.11–7.33 (m, 2H), 7.92 (dd, 2H, *J*_1_ = 16.0, *J*_2_ = 5.2 Hz). ^13^C NMR (CDCl_3_, 100 MHz): δ 22.3, 22.7, 24.4, 26.8, 29.2, 29.3, 29.6, 31.7, 31.9, 33.2, 36.4, 49.4, 52.1, 111.0, 114.9, 115.6, 117.8, 122.3, 124.4, 124.7, 126.5, 134.1, 134.6, 145.8, 151.3, 158.5, 162.4, 171.8. MS (ESI+) *m*/*z*: [M + H]^+^ calcd for C_29_H_35_ClN_2_O_3_, 495; found, 496.

#### 2-Hydroxy-6-(8-((1,2,3,4-tetrahydroacridin-9-yl)amino)octyl)benzoic Acid (**14**)

The title compound was obtained according to general procedure VI starting from **12**. The crude product was purified by filtration. Compound **14** was obtained as a white solid. Yield: 84%. HPLC purity: 95%. ^1^H NMR (CD_3_OD, 400 MHz): δ 1.21–1.43 (m, 8H), 1.52 (s, 2H), 1.76–1.83 (m, 2H), 1.93 (s, 4H), 2.67 (s, 2H), 2.88–2.94 (m, 2H), 2.99 (s, 2H), 3.92 (t, 2H, *J* = 6.4 Hz), 6.62 (dd, 2H, *J*_1_ = 18.5, *J*_2_ = 7.7 Hz), 7.11 (t, 1H, *J* = 7.8 Hz), 7.55 (t, 1H, *J* = 7.6 Hz), 7.74 (d, 1H, *J* = 8.4 Hz), 7.81 (t, 1H, *J* = 7.2 Hz), 8.35 (d, 1H, *J* = 8.7 Hz). ^13^C NMR (CD_3_OD, 100 MHz): δ 20.4, 21.5, 23.4, 26.0, 27.8, 28.5, 28.7, 29.0, 29.9, 31.2, 31.5, 34.9, 111.4, 113.9, 118.6, 121.0, 124.8, 125.0, 132.6, 137.7, 148.2, 154.8, 159.8. HRMS (ESI+) *m*/*z*: [M + H]^+^ calcd for C_28_H_34_N_2_O_3_, 447.26422; found, 447.26436.

#### 2-(8-((6-Chloro-1,2,3,4-tetrahydroacridin-9-yl)amino)octyl)-6-hydroxybenzoic Acid (**15**)

The title compound was obtained according to general procedure VI starting from **13**. The crude product was purified by filtration. Compound **15** was obtained as a white solid. Yield: 54%. HPLC purity: 95%. ^1^H NMR (CDCl_3_, 400 MHz): δ 1.41–1.25 (m, 5H), 1.53 (s, 1H), 1.79 (dd, 1H, *J*_1_ = 14.1, *J*_2_ = 6.8 Hz), 1.92 (d, 3H, *J* = 2.8 Hz), 2.64 (s, 1H), 2.89–2.77 (m, 1H), 2.97 (s, 1H), 3.31–3.24 (m, 1H), 3.91 (t, 2H, *J* = 7.3 Hz), 6.66 (dd, 2H, *J*_1_ = 13.1, *J*_2_ = 8.0 Hz), 7.17 (t, 1H, *J* = 7.8 Hz), 7.52 (dd, 1H, *J* = 9.3, *J*_2_ = 1.9 Hz), 7.75 (d, 1H, *J* = 1.9 Hz), 8.35 (d, 1H, *J* = 9.3 Hz). ^13^C NMR (CDCl_3_, 100 MHz): δ 20.4, 21.9, 24.1, 25.9, 27.8, 27.8, 28.4, 28.6, 29.6, 30.7, 31.8, 36.2, 48.3, 110.6, 111.7, 115.3, 118.5, 122.2, 125.5, 126.6, 134.0, 138.3, 138.4, 147.1, 151.6, 155.3, 163.1, 173.2. HRMS (ESI+) *m*/*z*: [M + H]^+^ calcd for C_28_H_33_ClN_2_O_33_, 481.22525; found, 481.20619.

#### 3-(8-((1,2,3,4-Tetrahydroacridin-9-yl)amino)octyl)phenol (**16**)

The title compound was obtained according to general procedure VII starting from **22**. The crude product was purified by column chromatography (9.5:0.5:0.1 dichloromethane/methanol/aqueous 32% ammonia). Compound **16** was obtained as a sticky yellow-brown oil. Yield: 41%. HPLC purity: 95%. ^1^H NMR (CDCl_3_, 400 MHz): δ 1.22–1.45 (m, 10H), 1.67 (q, 2H, *J* = 6.8 Hz), 1.87 (m, 4H), 2.44 (t, 2H, *J* = 7.6 Hz), 2.66 (m, 2H), 3.10 (m, 2H), 3.62 (t, 2H, *J* = 6.8 Hz), 4.44 (br, NH), 6.62 (m, 2H), 6.73 (d, 1H, *J* = 8.0 Hz), 7.07 (t, 1H, *J* = 8.0 Hz), 7.34 (t, 1H, *J* = 8.0 Hz), 7.53 (t, 1H, *J* = 8.0 Hz), 8.00–8.06 (m, 2H). ^13^C NMR (CDCl_3_, 100 MHz): δ 22.3, 22.7, 24.4, 26.4, 28.5, 28.6, 29.0, 29.6, 30.6, 31.3, 32.3, 35.4, 48.9, 113.0, 114.4, 115.3, 119.1, 119.5, 123.1, 123.8, 126.8, 129.1, 129.1, 144.1, 152.0, 157.1, 157.1. MS (ESI+) *m*/*z*: [M + H]^+^ calcd for C_27_H_34_N_2_O, 402; found, 403.

#### 3-(8-((6-Chloro-1,2,3,4-tetrahydroacridin-9-yl)amino)octyl)phenol (**17**)

The title compound was obtained according to general procedure VII starting from **23**. The crude product was purified by column chromatography (7:2.5:0.5:0.05 petroleum ether/ethyl acetate/methanol/aqueous 32% ammonia). Compound **17** was obtained as a sticky yellow-brown oil. Yield: 45%. HPLC purity: 97%. ^1^H NMR (CDCl_3_, 400 MHz): δ 1.40–1.13 (m, 8H), 1.51 (m, 2H), 1.69 (m, 4H), 2.00–1.84 (m, 2H), 2.55–2.43 (m, 2H), 2.73–2.63 (m, 2H), 3.17–3.02 (m, 2H), 3.64–3.51 (m, 2H), 4.36–4.00 (m, 1H), 6.70 (m, 3H), 7.12 (m, 1H), 7.28 (m, 1H), 7.97 (m, 2H). ^13^C NMR (CDCl_3_, 100 MHz): δ 22.3, 22.7, 24.3, 26.4, 28.6, 28.7, 29.1, 30.7, 31.4, 32.8, 35.5, 49.1, 112.9, 114.7, 115.4, 117.6, 119.7, 124.3, 124.8, 126.1, 129.2, 134.7, 144.3, 146.8, 151.7, 157.1, 158.6.

#### Methyl 2-(8-Hydroxyoctyl)-6-methoxybenzoate (**19a**)

The title compound was obtained according to general procedure II starting from **18a**. The crude product was purified by column chromatography (from 8:2 to 6.5:3.5 petroleum ether/ethyl acetate). Compound **19a** was obtained as a light brown oil. Yield: 60%. ^1^H NMR (CDCl_3_, 500 MHz): δ 1.30 (m, 8H, 3–6), 1.51–1.56 (m, 4H), 1.86 (s, 1H), 2.52 (t, 2H, *J* = 7.0 Hz), 2.61 (t, 2H, *J* = 6.0 Hz), 3.80 (s, 3H), 3.90 (s, 3H), 6.75 (d, 1H, *J* = 8.2 Hz), 6.81 (d, 1H, *J* = 7.6 Hz), 7.26 (t, 1H, *J* = 8.0 Hz). ^13^C NMR (CDCl_3_, 125 MHz): δ 25.8, 29.4–29.5, 31.2, 32.8, 33.6, 52.3, 56.0, 63.1, 108.5, 121.6, 123.5, 130.4, 141.4, 156.4, 169.1.

#### 3-Methoxyphenyloctan-1-ol (**19b**)

The title compound was obtained according to general procedure II starting from **18b**. The crude product was purified by column chromatography (from 8:2 to 6.5:3.5 petroleum ether/ethyl acetate). Compound **19b** was obtained as a light yellow oil. Yield: 70%. ^1^H NMR (CDCl_3_, 300 MHz): δ 1.33 (m, 8H), 1.54–1.58 (m, 2H), 1.60–1.63 (m, 2H), 2.59 (t, 2H, *J* = 6.0 Hz), 3.64 (t, 2H, *J* = 6.0 Hz), 3.81 (s, 3H), 6.72–6.79 (m, 3H), 7.18–7.21 (m, 1H). ^13^C NMR (CDCl_3_, 75 MHz): δ 25.9, 29.4–29.6, 31.5, 32.9, 36.2, 55.3, 63.2, 110.9, 114.4, 121.1, 129.3, 144.7, 159.7.

#### 8-(3,5-Dimethoxyphenyl)octan-1-ol (**19c**)

The title compound was obtained according to general procedure II starting from **18c**. The crude product was purified by column chromatography (from 8:2 to 6.5:3.5 petroleum ether/ethyl acetate). Compound **19c** was obtained as a light brown oil. Yield: 60%. ^1^H NMR (CDCl_3_, 300 MHz): δ 1.32 (s, 8H), 1.53–1.60 (m, 4H), 2.07 (s, 1H), 2.54 (t, 2H, *J* = 7.7 Hz), 3.62 (t, 2H, *J* = 6.6 Hz), 3.78 (s, 6H), 6.30 (t, 1H, *J* = 2.2 Hz), 6.35 (d, 2H, *J* = 2.2 Hz). ^13^C NMR (CDCl_3_, 75 MHz): δ 25.8, 29.3–29.6, 31.3, 32.8, 36.4, 55.3, 63.1, 97.7, 106.6, 145.4, 160.8.

#### Synthesis of 8-(3-Hydroxyphenyl)octan-1-ol (**19d**)

Mixture of cardanol **2** (2 g, ∼6.6 mmol), distilled acetic anhydride (1.25 mL, 13.1 mmol), and phosphoric acid (4 drops) were heated in a conventional microwave oven for 3 min (3 × 1 min) at a power of 450 W (50%). Then, the mixture was extracted with ethyl acetate (3 × 10 mL) and the combined organic layers were washed with 5% sodium bicarbonate solution (10 mL), 10% hydrochloric acid solution (10 mL), and saturated saline (10 mL) and dried over anhydrous Na_2_SO_4_. After evaporating the solvent under reduced pressure, the reaction mixture was purified by silica gel column chromatography (dichloromethane, 100%), providing the acetylated mixture intermediates in 90% yield. The mixture of acetylated cardanols (2 g, ∼9.4 mmol) was solubilized in dichloromethane/methanol (1:1, 60 mL). The reaction system was cooled at 0 °C, and the solution was treated with ozone for approximately 3 h. After that, the excess of ozone was purged with the nitrogen flow and the solution was transferred to another flask. The mixture was dissolved in methanol/ethanol (1:1, 60 mL) and cooled at 0 °C under an ice–water bath, and sodium borohydride (2 g, 8.0 equiv) was added. The reaction was left warming to rt under vigorous stirring for 16 h. Then, the mixture was acidified to pH 3.0 with concentrated hydrochloric acid solution and the residue was extracted with ethyl acetate (3 × 20 mL). The combined organic fractions were washed with saturated sodium chloride solution (10 mL), dried over anhydrous Na_2_SO_4_, and evaporated. The product was purified by silica gel column chromatography (dichloromethane/ethanol, 0–5%), giving **19d** as a light yellow oil. Yield: 67%. ^1^H NMR (CDCl_3_, 500 MHz): δ 1.30 (s, 8H), 1.56–1.58 (m, 4H), 2.53 (t, 2H, *J* = 7.5 Hz), 3.65 (t, 2H, *J* = 6.5 Hz), 4.11 (s, 2H), 6.66 (d, 1H, *J* = 8.3 Hz), 6.67 (s, 1H), 6.72 (d, 1H, *J* = 7.4 Hz), 7.12 (t, 1H, *J* = 7.6 Hz). ^13^C NMR (CDCl_3_, 125 MHz): δ 25.8, 29.2, 29.3, 29.4, 31.3, 32.7, 35.9, 63.3, 112.8, 115.6, 120.8, 129.5, 144.9, 156.0.

#### Synthesis of 8-(3-(Benzyloxy)phenyl)octan-1-ol (**20**)

Compound **19d** (0.74 mmol, 1.0 equiv) and K_2_CO_3_ (1.11 mmol, 1.5 equiv) were stirred in acetone (7.40 mL). Benzyl bromide (0.74 mmol, 1.0 equiv) was added, and the reaction was stirred at reflux temperature for 12 h. The solvent was then removed in vacuo, and the residue was dissolved in ethyl acetate and washed with water. The organic layers were combined, dried over anhydrous Na_2_SO_4_, and concentrated in vacuo. The crude product was purified by column chromatography (7.5:2:0.5 petroleum ether/ethyl acetate/methanol). Compound **20** was obtained as a colorless oil. Yield: 92%. ^1^H NMR (CDCl_3_, 400 MHz): δ 1.36 (s, 8H), 1.56–1.66 (m, 4H), 1.94 (s, OH), 2.62 (t, 2H, *J* = 7.6 Hz), 3.63 (t, 2H, *J* = 6.8 Hz), 5.07 (s, 2H), 6.87–6.82 (m, 3H), 7.22 (s, 1H, *J* = 8 Hz), 7.35–7.48 (m, 5H). ^13^C NMR (CDCl_3_, 100 MHz): δ 25.7, 29.2, 29.4, 29.5, 31.3, 32.7, 36.0, 62.9, 69.9, 111.7, 115.2, 121.2, 127.5, 127.9, 128.5, 129.2, 137.2, 144.6, 158.8.

#### Methyl 2-Methoxy-6-(8-((methylsulfonyl)oxy)octyl)benzoate (**21a**)

The title compound was obtained according to general procedure III starting from **19a**. The crude product was purified by column chromatography (100% dichloromethane). Compound **21a** was obtained as a colorless oil. Yield: 85%. ^1^H NMR (CDCl_3_, 500 MHz): δ 1.30 (m, 6H), 1.38 (m, 2H), 1.57 (m, 2H), 1.70–1.76 (m, 2H), 2.53 (t, 2H, *J* = 7.8 Hz), 3.00 (s, 3H), 3.81 (s, 3H), 3.90 (s, 3H), 4.21 (t, 2H, *J* = 6.0 Hz), 6.76 (d, 1H, *J* = 8.3 Hz), 6.81 (d, 1H, *J* = 7.7 Hz), 7.27 (t, 1H, *J* = 8.0 Hz). ^13^C NMR (CDCl_3_, 125 MHz): δ 25.5, 29.0, 29.2–29.4, 31.2, 33.6, 37.5, 52.3, 56.0, 70.3, 108.5, 121.6, 123.6, 130.4, 141.3, 156.4, 169.1.

#### 8-(3-Methoxyphenyl)octyl Methanesulfonate (**21b**)

The title compound was obtained according to general procedure III starting from **19b**. The crude product was purified by column chromatography (100% dichloromethane). Compound **21b** was obtained as a light brown oil. Yield: 60%. ^1^H NMR (CDCl_3_, 300 MHz): δ 1.32–1.42 (m, 8H), 1.56–1.63 (m, 2H), 1.79–1.89 (m, 2H), 2.57 (t, 2H, *J* = 6.0 Hz), 2.98 (s, 3H), 3.40 (t, 2H, *J* = 6.0 Hz), 3.79 (s, 3H), 6.71–6.78 (m, 3H), 7.20 (dd, 1H, *J* = 9.0 Hz). ^13^C NMR (CDCl_3_, 75 MHz): δ 28.2, 28.7–29.3, 31.3, 32.8, 34.1, 36.0, 37.5, 55.1, 110.8, 114.2, 120.8, 129.2, 144.5, 159.5.

#### 8-(3,5-Dimethoxyphenyl)octyl Methanesulfonate (**21c**)

The title compound was obtained according to general procedure III starting from **19c**. The crude product was purified by column chromatography (100% dichloromethane). Compound **21c** was obtained as a light brown oil. Yield: 80%. ^1^H NMR (CDCl_3_, 300 MHz): δ 1.33–1.44 (s, 8H), 1.60 (t, 2H, *J* = 6.5 Hz), 1.69–1.78 (m, 2H), 2.54 (t, 2H, *J* = 7.6 Hz), 2.99 (s, 3H), 3.78 (s, 6H), 4.21 (t, 2H, *J* = 6.5 Hz), 6.30 (t, 1H, *J* = 2.2 Hz), 6.34 (d, 2H, *J* = 2.2 Hz). ^13^C NMR (CDCl_3_, 75 MHz): δ 25.5, 29.0, 29.2–29.4, 31.3, 36.3, 37.4, 55.3, 70.3, 97.7, 106.6, 145.3, 160.8.

#### 8-(3-(Benzyloxy)phenyl)octyl Methyl Sulfate (**21d**)

The title compound was obtained according to general procedure III starting from **20**. The crude product was purified by column chromatography (6.5:3.5 dichloromethane/petroleum ether). Compound **21d** was obtained as a colorless oil. Yield: 87%. ^1^H NMR (CDCl_3_, 400 MHz): δ 1.35–1.41 (m, 8H), 1.64–1.76 (m, 4H), 2.61 (t, 2H, *J* = 7.6 Hz), 2.96 (s, 3H), 4.21 (t, 2H, *J* = 6.0 Hz), 5.06 (s, 2H), 6.81–6.85 (m, 3H), 7.22 (t, 1H, *J* = 8.0 Hz), 7.34–7.47 (m, 5H). ^13^C NMR (CDCl_3_, 100 MHz): δ 25.4, 28.9, 29.1, 29.1, 29.29, 31.2, 35.9, 37.2, 69.8, 70.2, 111.7, 115.2, 121.1, 127.5, 127.9, 128.5, 129.2, 137.2, 144.5, 158.8.

#### *N*-(8-(3-(Benzyloxy)phenyl)octyl)-1,2,3,4-tetrahydroacridin-9-amine (**22**)

The title compound was obtained according to general procedure IV using **4a** and **21d**. The crude product was purified by column chromatography (6.5:3:0.5 petroleum ether/ethyl acetate/NEt_3_). Compound **22** was obtained as a sticky yellow-brown oil. Yield: 32%. ^1^H NMR (CDCl_3_, 400 MHz): δ 1.30–1.39 (m, 8H), 1.56–1.89 (m, 4H), 1.89 (m, 4H), 2.55 (t, 2H, *J* = 7.6 Hz), 2.62 (m, 2H), 3.14 (m, 2H), 3.61 (t, 2H, *J* = 7.2 Hz), 5.02 (s, 2H), 6.75–6.79 (m, 3H), 7.17 (t, 1H, *J* = 7.6 Hz), 7.30–7.42 (m, 6H), 7.53 (t, 1H, *J* = 7.2 Hz), 8.00 (d, 1H, *J* = 8.4 Hz), 8.12 (d, 1H, *J* = 8.00 Hz). ^13^C NMR (CDCl_3_, 100 MHz): δ 22.7, 23.0, 24.7, 26.9, 29.1, 29.2, 29.3, 31.2, 31.7, 33.8, 35.9, 49.5, 69.8, 111.6, 115.1, 115.6, 120.0, 121.1, 122.9, 123.5, 127.5, 127.8, 128.3, 128.5, 129.1, 137.1, 144.4, 147.2, 150.9, 158.2, 158.8.

#### *N*-(8-(3-(Benzyloxy)phenyl)octyl)-6-chloro-1,2,3,4-tetrahydroacridin-9-amine (**23**)

The title compound was obtained according to general procedure IV using **4b** and **21d**. The crude product was purified by column chromatography (7:2:0.5 petroleum ether/ethyl acetate/NEt_3_). Compound **23** was obtained as a sticky yellow-brown oil. Yield: 24%. HPLC purity: 95%. ^1^H NMR (CDCl_3_, 400 MHz): δ 1.36–1.24 (m, 8H), 1.64–1.59 (m, 4H), 1.91–1.88 (t, 4H, *J* = 8 Hz), 2.56 (t, 2H, *J* = 8 Hz), 2.63 (t, 2H, *J* = 8 Hz), 3.02 (t, 2H, *J* = 8 Hz), 3.47 (t, 2H, *J* = 8164 Hz), 5.03 (s, 2H), 6.81–6.76 (m, 3H), 7.43–7.15 (m, 8H), 7.90–7.87 (m, 2H). ^13^C NMR (CDCl_3_, 100 MHz): δ 22.5, 22.8, 24.4, 26.8, 29.1, 29.2, 29.3, 31.2, 31.7, 33.8, 35.9, 49.5, 69.8, 111.6, 115.2, 121.1, 124.2, 124.6, 127.4, 127.8, 128.5, 129.1, 137.1, 144.4, 151.1, 158.8.

### Crystallization of Human BChE in Complex with **5**

Recombinant 4sugOff/L530Stop human BChE (hBChE) was produced in Chinese hamster ovary cells^80^ and purified by hBChE specific affinity chromatography (Hupresin; CHEMFORASE, Rouen, France) and size exclusion chromatography (Superdex 200, GE Healthcare) as previously described.^81^ The stock solution of compound **5** (100 mM) was prepared in MeOH. Crystallization was carried out at 293 K with the hanging drop vapor diffusion method using a 6.5 mg/mL hBChE solution and a mother liquor containing 1 mM ligand, of composition 1% MeOH, and 2.15 M (NH_4_)_2_SO_4_ in 100 mM 2-(*N*-morpholino)ethanesulfonic acid (MES) (pH 6.5) buffer. Large crystals (200 to 400 μM) grew in about a week. Crystals were cryoprotected in a solution of 0.1 M MES (pH 6.5), 2.15 M (NH_4_)_2_SO_4_, 20% glycerol, 1 mM ligand, and 1% MeOH before flash cooling into liquid nitrogen.

### Structure Determination of Human BChE in Complex with **5**

X-ray diffraction data were collected at synchrotron SOLEIL (Saint Aubin, France) at the PROXIMA-2 beamline at 100 K. Images from two isomorphous crystals of equal quality recorded on an EIGER 16M detector were processed with the XDS suite of software.^82^ The structure resolution and refinement were realized using the PHENIX software suite.^83^ An initial model was obtained by molecular replacement using Phaser-MR^84^ included in PHENIX and the hBChE structure (PDB entry: 1p0i) devoid of any ligands, glycans, or water molecules. Extra electron density was observed close to the active-site gorge that allowed modeling of compound **5**. Ligand geometry restraints were calculated using PHENIX eLBOW^85^ included in PHENIX and the semiempirical quantum mechanical method (AM1). The model was refined by iterative cycles of phenix.refine and model building using Coot.^86^ Coordinates and structure factors of the hBChE–**5** complex are deposited into the Protein Data Bank under accession code 7bgc. Data collection and refinement statistics, as calculated using PHENIX, are shown in Table S2. The protein structures were illustrated using the program PyMOL (Schrodinger LLC).

### Biology

#### Inhibition of Human AChE and BChE Activities

The method of Ellman et al. was followed.^38^ AChE stock solution was prepared by dissolving human recombinant AChE lyophilized powder (Sigma, Italy) in 100 mM phosphate buffer (pH = 8.0) containing 0.1% Triton X-100. Stock solution of BChE from human serum (Sigma, Italy) was prepared by dissolving the lyophilized powder in an aqueous solution of 0.1% gelatine. Stock solutions of tested compounds (1 mM) were prepared in methanol and diluted in methanol. The assay solution consisted of a 0.1 M phosphate buffer (pH 8.0), with the addition of 340 μM 5,5′-dithio-bis(2-nitrobenzoic acid), 0.02 units of hAChE or hBChE, and 550 μM substrate (acetylthiocoline iodide or butyrylthiocholine iodide, respectively). Assays were done with a blank containing all components except the enzyme to account for nonenzymatic substrate hydrolysis. Tested tacrine hybrids were added to the assay solution and preincubated with the enzyme for 20 min before the addition of the substrate. Initial rate assays were performed at 37 °C with a JASCO V-530 double-beam spectrophotometer (JASCO Europe, Italy) equipped with a thermostated cuvette holder (37 °C). The absorbance value at 412 nm was recorded for 240 s, and enzyme activity was calculated from the slope of the obtained linear trend. The reaction rates obtained in the presence and in the absence of the tested compound were compared, and the percent inhibition was calculated. Five different concentrations of each compound were used to obtain inhibition of enzyme activity between 20 and 80%. IC_50_ values were determined graphically from log concentration–inhibition curves (GraphPad Prism 4.03 software, GraphPad Software Inc.). Each final value is the mean of at least two independent experiments each performed in triplicate.

#### Determination of Hepatotoxicity of **5**–**17** on HepG2 Cells

HepG2 cells (human hepatocytes from liver carcinoma, American Type Culture Collection, ATCC) were grown in the DMEM supplemented with 10% FBS and 50 units/mL penicillin/streptomycin (Life Technologies Italia, Monza, MB, Italy) at 37 °C in a humidified atmosphere containing 5% CO_2_. For the experiments, cells (0.3 × 10^5^ cells/well) were seeded in a 96-well plate in a complete medium; after 24 h, the medium was removed, and cells were exposed to the increasing concentrations of compounds **5**–**17**, reference compounds (0.1, 1, and 10 μM), or vehicle and dissolved in the complete DMEM for 24 h. Cell viability was measured by the MTT assay.

#### Determination of Toxicity and Activity Profiles of Selected Compounds on SH-SY5Y and BV-2 Cells

##### Chemicals and Reagents

High-glucose Dulbecco’s modified Eagle’s medium (DMEM), l-glutamine solution, penicillin/streptomycin, trypsin–EDTA solution, phosphate-buffered saline (PBS), LPS from *Escherichia coli* serotype O127:B8, all-*trans*-retinoic acid (RA), dimethyl sulfoxide (DMSO), 3-(4,5-dimethylthiazol-2-yl)-2,5-diphenyl tetrazolium bromide (MTT), and primers for RT-PCR were purchased from Sigma-Aldrich–Merck (Milan, Italy). Fetal bovine serum (FBS) and low-endotoxin FBS were purchased from Euroclone (Milan, Italy).

##### Cell Cultures and Treatments

SH-SY5Y cell line was purchased from Sigma-Aldrich–Merck (ECACC 94030304) (Milan, Italy) and was grown in a high-glucose DMEM supplemented with 10% (v/v) FBS, 2 mM l-glutamine, 50 U/mL penicillin, and 50 μg/mL streptomycin, as previously reported.^87^ Before starting the experiments, cells were differentiated with all-*trans*-retinoic acid (10 μM) for 7 days. Differentiated SH-SY5Y cells were treated for 24 h with various concentrations of the tested compounds. BV-2 murine microglial cells were kindly provided by Prof. Elisabetta Blasi (University of Modena and Reggio Emilia, Modena, Italy) and were grown in a high-glucose DMEM supplemented with 10% (v/v) low-endotoxin FBS, 2 mM l-glutamine, 50 U/mL penicillin, and 50 μg/mL streptomycin. BV-2 cells were treated for 24 h with various concentrations of the tested compounds and then exposed to LPS (100 ng/mL) for further 24 h.

##### MTT Viability Assay

Cell viability was evaluated by measuring MTT reduction as reported in ref (88). The cells were seeded in 96-well tissue culture plates, and at the end of treatments, cells were incubated with 0.5 mg/mL MTT solution for 30 min (BV-2 cells) or 90 min (SH-SY5Y cells). At the end, the MTT solution was replaced with DMSO in order to solubilize the formed formazan crystals. Finally, formazan formation was measured spectrophotometrically at 595 nm using a microplate spectrophotometer (VICTOR3 V Multilabel Counter; PerkinElmer, Wellesley, MA, USA). Data are expressed as a percentage of control cells, which are considered as 100% cell viability.

##### RNA Extraction

The extraction of total RNA was conducted using an RNeasy Mini Kit (QIAGEN GmbH, Hilden, Germany), following the manufacturer’s protocol. The yield and purity of the RNA were measured using a NanoVue spectrophotometer (GE Healthcare, Milan, Italy).

##### Real-Time Polymerase Chain Reaction (PCR)

One microgram of total RNA was reverse-transcribed to cDNA using an iScript cDNA Synthesis Kit (Bio-Rad, Hercules, CA, USA), following the manufacturer’s instructions. The real-time PCR was carried out in a total volume of 10 μL containing 2.5 μL (12.5 ng) of cDNA, 5 μL of SsoAdvanced Universal SYBR Green Supermix (Bio-Rad), and 0.5 μL (500 nM) of each primer. IL-1β, TNF-α, iNOS, and COX-2 (Sigma-Aldrich–Merck, Milan, Italy) expression levels were evaluated, while as the reference gene for BV-2 cells was used GAPDH. The primer sequences are reported in Table S3. The following protocol was followed to amplify the cDNA: 30 s at 95 °C (to activate the polymerase) followed by 5 s at 95 °C and 30 s at 60 °C for 40 cycles. Normalized expression levels were evaluated in respect to control cells according to the 2^–ΔΔCT^ method.

##### Immunofluorescence Confocal Microscopy

BV-2 cells were seeded directly on glass coverslips in 6-well plates. At the end of treatments, cells were fixed at room temperature with paraformaldehyde 2% for 15 min and then permeabilized with Triton X-100 0.1% for 10 min. Subsequently, BV-2 cells were incubated overnight with a polyclonal antibody (1:500) against NF-κB p65. After PBS extensive washing, cells were exposed, for 1 h at room temperature, to a secondary Alexa Fluor 488-conjugated antirabbit IgG antibody (1:1000). Nuclei were stained with 1 μg/mL 4′-6-diamidino-2-phenylindole (DAPI). Slides were analyzed with a C2 Plus confocal laser scanning microscope (Nikon Instruments, Firenze, Italy). Images were processed using NIS-Elements imaging software (Nikon Instruments, Firenze, Italy).

##### IL-1β Quantification

BV-2 cells were seeded in 6-well plates, and at the end of treatments, the culture media were taken to detect IL-1β concentration. IL-1β quantification was performed using an IL-1β ELISA Kit following the manufacturer’s instructions (Sigma-Aldrich–Merck). Absorbance (450 nm) was measured using a microplate spectrophotometer (VICTOR3 V Multilabel Counter).

##### Statistical Analysis

All the analyses were carried out at least in triplicate, and data were expressed as mean ± standard error. To compare differences among groups, one-way ANOVA followed by Dunnett’s or Tukey’s test (Prism 7; GraphPad Software, San Diego, CA) was used. Differences at the level of *p* < 0.05 were considered statistically significant.

##### PAMPA Assay

The filter membrane of the donor plate was coated with PBL (polar brain lipid, Avanti, AL, USA) in dodecane (4 μL of 20 mg/mL PBL in dodecane), and the acceptor well was filled with 300 μL of PBS (pH 7.4) buffer (*V*_A_). Tested compounds were dissolved first in DMSO and diluted with PBS (pH 7.4) to reach the final concentration in the donor well (40–100 μM). Concentration of DMSO did not exceed 0.5% (v/v) in the donor solution. Three hundred microliters of the donor solution was added to the donor wells (*V*_D_), and the donor filter plate was carefully put on the acceptor plate so that the coated membrane was “in touch” with both donor solution and acceptor buffer. The test compound diffused from the donor well through the lipid membrane (area = 0.28 cm^2^) to the acceptor well. The concentrations of the drug in both donor and acceptor wells were assessed after 3, 4, 5, and 6 h of incubation in quadruplicate using the UV plate reader Synergy HT (Biotek, Winooski, VT, USA) at the maximum absorption wavelength of each compound. In addition to that, solution of theoretical compound concentration, simulating the equilibrium state established if the membrane was ideally permeable, was prepared and assessed as well. Concentrations of the compounds in the donor and acceptor wells and equilibrium concentration were calculated from the standard curve and expressed as the permeability (*P*_e_) according to the equation^89^
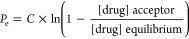
where 

##### Plasma Stability Assay

Stability of **5** in plasma was assessed by incubating the compound over a 6 h time frame at 37 °C and analyzing its concentration by high-performance liquid chromatography (HPLC) coupled with mass spectrometry (MS). Pooled human plasma was purchased from VWR (Stříbrná Skalice, Czech Republic). Stock solution of **5** in DMSO was diluted with plasma to reach a 10 μM final concentration of **5**. Two hundred microliters of 10 μM plasma solution was pipetted to mictrotubes. The first sample was taken to be extracted, while the others were incubated at 37 °C. Every hour, one sample was taken and extracted. Ten microliters of internal standard (IS; 200 μM tacrine-trolox hybrid described by Nepovimova et al. in methanol)^90^ was added to every sample, vortexed, and supplemented by 700 μL of LC–MS grade acetonitrile (VWR, Stříbrná Skalice, Czech Republic). Then, the sample was shaken (3 min, level 7, VM-10 Witeg, Wertheim, Germany) and centrifuged (10,000 RPM, 2 min, Roth Gusto, ROTILABO, Illinois, USA) and 700 μL of supernatant was transferred to the vial and analyzed by HPLC–MS. Calibration samples were prepared by adding 10 μL of 5 (20–400 μM in DMSO) to 190 μL of blank plasma; final concentrations were in a range of 1–20 μM. Then, 10 μL of IS was added and samples were extracted as above and analyzed by HPLC–MS.

HPLC–MS analysis was performed using Dionex UltiMate 3000 UHPLC consisting of an RS LPG quaternary pump, RS column compartment, RS autosampler, and diode array detector controlled by Chromeleon (version 7.2.9 build 11323) software (Thermo Fisher Scientific, Germering, Germany) with a Q Exactive Plus Orbitrap mass spectrometer with Thermo Xcalibur (version 3.1.66.10.) software (Thermo Fisher Scientific, Bremen, Germany). Detection was performed by mass spectrometry in positive mode. Settings of the heated electrospray source were as follows: spray voltage, 3.5 kV; capillary temperature, 300 °C; sheath gas, 55 arbitrary units; auxiliary gas, 15 arbitrary units; spare gas, 3 arbitrary units; probe heater temperature, 250 °C; max spray current, 100 μA; and S-lens RF level: 50. The concentration assessment of **5** was performed in reverse-phase gradient mode using a Kinetex EVO C18 column (2.1 × 50 mm, 1.7 μm, Phenomenex, Torrance, California, USA) with a Kinetex SecurityGuard Ultra C18 guard column (2.1 mm, Phenomenex, Torrance, California, USA). Mobile phase A was ultrapure water of ASTM I type (resistance, 18.2 MΩ cm at 25 °C) prepared by Barnstead Smart2Pure 3 UV/UF apparatus (Thermo Fisher Scientific, Bremen, Germany) with 0.1% (v/v) formic acid (LC–MS grade, VWR, Stříbrná Skalice, Czech Republic); mobile phase B was acetonitrile (LC–MS grade, VWR, Stříbrná Skalice, Czech Republic) with 0.1% (v/v) of formic acid. The column was tempered to 35 °C, mobile phase flow was set to 0.5 mL/min, and injection volume was 5 μL. The method started with 5% B and was steady for 0.3 min, and then the gradient went from 5 to 100% B in 3 min, was kept at 100% B for 0.5 min, then went back to 5% B, and equilibrated for 2.2 min. Total runtime of the method was 6 min. The compound and IS were detected with a mass spectrometer in the total ion current scan in a range of 105–700 *m*/*z* in positive mode. Retention time for **5** was 3.32 min with mass searched 475.2952, and retention time for IS was 3.29 min with mass searched 592.3297. Calibration had five points (1, 5, 10, 15, and 20 μM) and was linear along the entire range.

## Supporting Information Available

Physicochemical parameter evaluation of **1a**; NMR spectra, chromatograms, and MS spectra for compounds **5** and **6**; experimental details for crystallography; cytotoxicity on SH-SY5Y and BV-2 cells; and primer sequences (PDF)
Molecular formula strings and biological data (CSV)

## Notes

The structural data of the hBChE–**5** complex were deposited in the Protein Data Bank under accession number 7bgc. Authors will release the atomic coordinates and experimental data upon article publication.

## Abbreviations

Aβ: amyloid β
AChE: acetylcholinesterase
AChEI: acetylcholinesterase inhibitor
AD: Alzheimer’s disease
BChE: butyrylcholinesterase
CNS: central nervous system
CNSL: cashew nutshell liquid
hBChE: human butyrylcholinesterase
HDAC: histone deacetylase
IL-1β: interleukin-1β
iNOS: inducible nitric oxide synthase
LPS: lipopolysaccharide
MAPK: mitogen-activated protein kinase
MTDL: multi-target-directed ligand
MW: microwave
NF-κB: nuclear factor kappa B
PAMPA: parallel artificial membrane permeability assay
*P*_e_: effective permeability
RT-PCR: real-time polymerase chain reaction
SAR: structure–activity relationships
TLR4: Toll-like receptor 4
TNF-α: tumor necrosis factor-α
