# Supplementary material for: Sustainable Drug Discovery of Multi-Target-Directed Ligands for Alzheimer’s Disease
Source: J Med Chem. 2021 Apr 8;64(8):4972–90. doi: 10.1021/acs.jmedchem.1c00048 (PMC8154578; doi:10.1021/acs.jmedchem.1c00048)
Supplement: Supplementary file 1 — jm1c00048_si_001.pdf [file jm1c00048_si_001.pdf]

SUPPORTING INFORMATION

Sustainable Drug Discovery of Multi-Target-Directed Ligands for Alzheimer's Disease

Michele Rossi[†,○], *Michela Freschi*[‡,○], *Luciana de Camargo Nascente*[§,○], *Alessandra Salerno*[†], *Sarah de Melo Viana Teixeira*[§], *Florian Nachon*[∥], *Fabien Chantegreil*[∥], *Ondrej Soukup*[⊥,#], *Lukáš Prchal*[⊥], *Marco Malaguti*[‡], *Christian Bergamini*[†], *Manuela Bartolini*[†], *Cristina Angeloni*[∇], *Silvana Hrelia*[‡], *Luiz Antonio Soares Romeiro*[§], *and Maria Laura Bolognesi*[*,†]

[†]Department of Pharmacy and Biotechnology, Alma Mater Studiorum - University of Bologna, Via Belmeloro 6, 40126 Bologna, Italy

[‡]Department for Life Quality Studies, Alma Mater Studiorum - University of Bologna, Corso d'Augusto 237, 47921 Rimini, Italy

[§]Department of Pharmacy, Health Sciences Faculty, University of Brasília, Campus Universitário Darcy Ribeiro, 70910-900 Brasília, DF, Brazil

[∥]Département de Toxicologie et Risques Chimiques, Institut de Recherche Biomédicale des Armées, 91220 Brétigny-sur-Orge, France

[⊥]Biomedical Research Center, University Hospital, Sokolska 581, 500 05 Hradec Kralove, Czech Republic.

[#]Department of Toxicology and Military Pharmacy, Faculty of Military Health Sciences, University of Defence, Trebesska 1575, 500 01 Hradec Kralove, Czech Republic

[∇]School of Pharmacy, University of Camerino, Via Madonna delle Carceri 9, 62032 Camerino, (MC), Italy

*Email: marialaura.bolognesi@unibo.it

TABLE OF CONTENTS

Table S1. Physicochemical parameters evaluation of 1a…………….……………………………S3

Compound Purity and HPLC traces………………………………………………………..S3-S4

^{1}H-NMR and ^{13}C-NMR spectra of 5 and 6…………………….……………………..S5-S6

HRMS of 5 and 6…………………………………………………………………………...S7

Crystallization, Data Collection and Processing……………………………………..........S8

Table S2. Data collection and refinement statistics……………….……………………S8-S9

Figure S1. Neurotoxicity of 5, 6, 9, 12-15 and 17 on SH-SY5Y cells……………………………S10

Figure S2. Cytotoxicity of 5, 6, 9, 12 - 15 and 17 on murine microglial BV-2 cells…………..S10

Table S3.BV-2 cell primers for real-time PCR……………………..……………………….S11

Bibliography…………………………………………………………………………...S11

Preliminary physicochemical parameters evaluation of 1a.

Method: The aqueous acid ionization constants pK_a and the octanol−water partition coefficient (log*P*) were measured by automated potentiometric titration using the instrument SiriusT3 (Sirius Analytical, Forest Row, East Sussex, U.K.).[1]

Table S1: Preliminary physicochemical parameters of compound **1a.** BBB penetration estimation and log *P* were not determined (N.D.) because of biased results due to the **1a** low solubility in the assay.

**Compound**	**BBB penetration estimation *Pe* ± SEM (× 10^{-6}cm s^{-1})**	**p*K*a ± SD**	**Log *P* ± SD**
**1a**	N.D.	4.10 ± 0.06	N.D.

Compound Purity and HPLC traces.

Method: compounds purity was determined using Kinetex® 5µm EVO C18 100 Å, LC Column 150 x 4.6 mm and a HPLC Jasco Corporation (Tokyo, Japan) instrument, model PU-1580 UV equipped with 20 µL loop valve. HPLC parameters were the following: ACN/H_2O/trifluoroacetic acid 45/55/0.05%; flow rate: 1 mL/min; elution type: isocratic; detection UV-Vis Abs at 254 nm. The samples were dissolved in ACN/H_2O/trifluoroacetic acid 45/55/0.05%

<u>**Compound 5**</u>

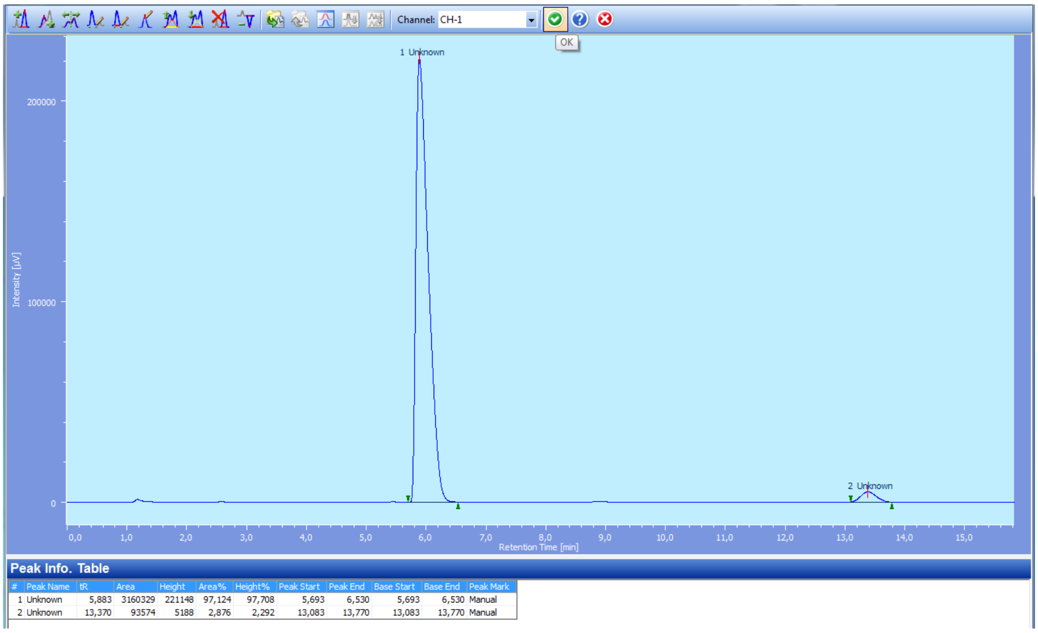

#	Peak Name	tR	Area	Height	Area%	Height%	Peak Start	Peak End	Base Start	Base End	Peak Mark
1	Unknown	5,883	3160329	221148	97,124	97,708	5,693	6,530	5,693	6,530	Manual
2	Unknown	13,370	93574	5188	2,876	2,292	13,083	13,770	13,083	13,770	Manual

Compound 6

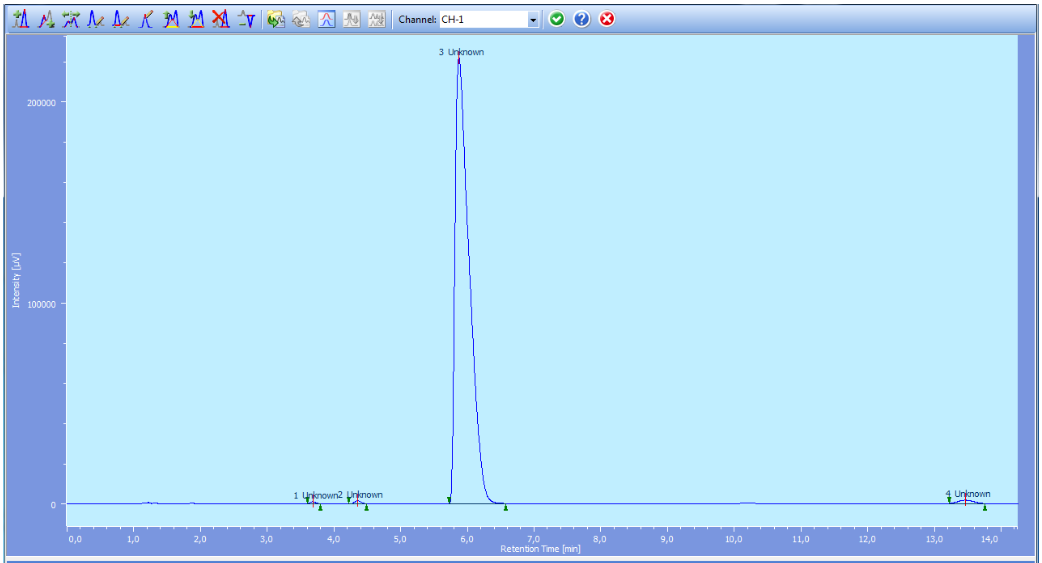

Peak Info. Table

#	Peak Name	tR	Area	Height	Area%	Height%	Peak Start	Peak End	Base Start	Base End	Peak Mark
1	Unknown	3,680	5787	1015	0,176	0,450	3,600	3,793	3,600	3,793	Manual
2	Unknown	4,353	10191	1507	0,309	0,668	4,220	4,487	4,220	4,487	Manual
3	Unknown	5,870	3254034	221331	98,680	98,125	5,720	6,570	5,720	6,570	Manual
4	Unknown	13,453	27564	1707	0,836	0,757	13,213	13,747	13,213	13,747	Manual

^{1}H-NMR and ^{13}C-NMR spectra (400 MHz, $CDCl_3$)

<u>Compound 5</u>

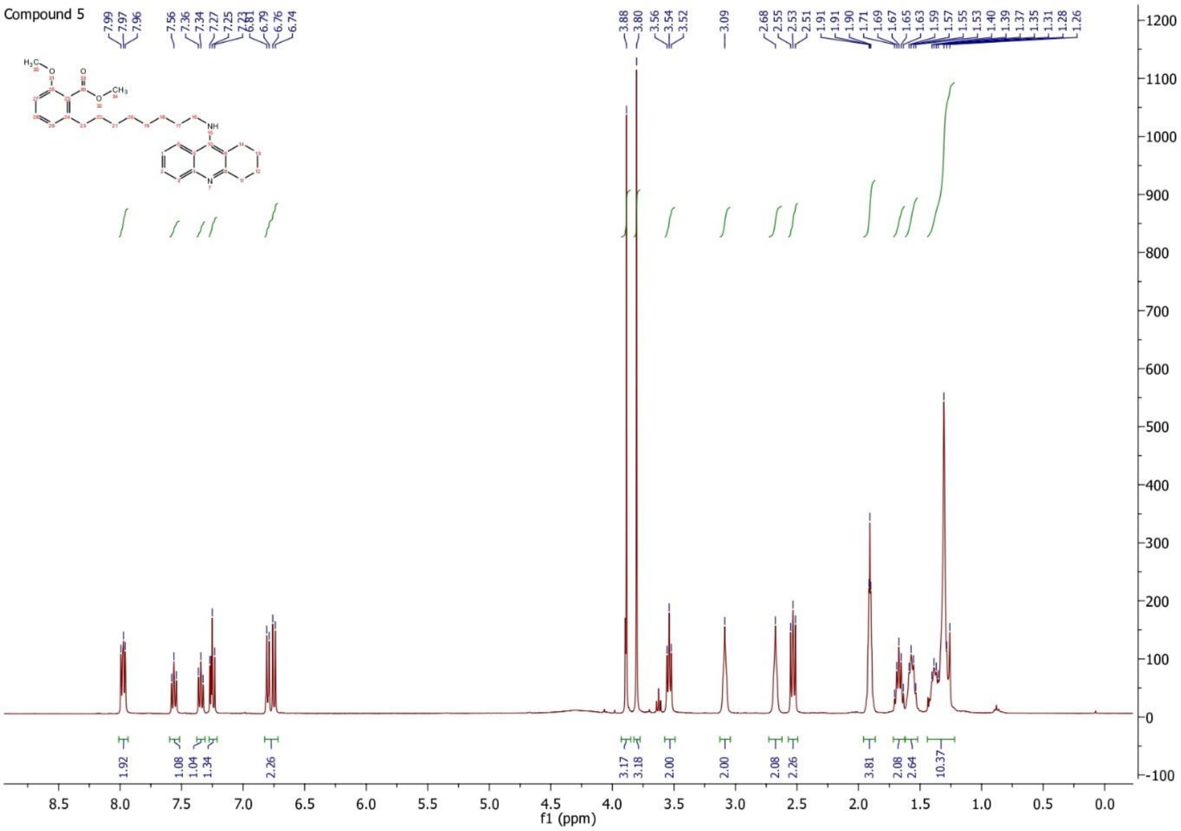

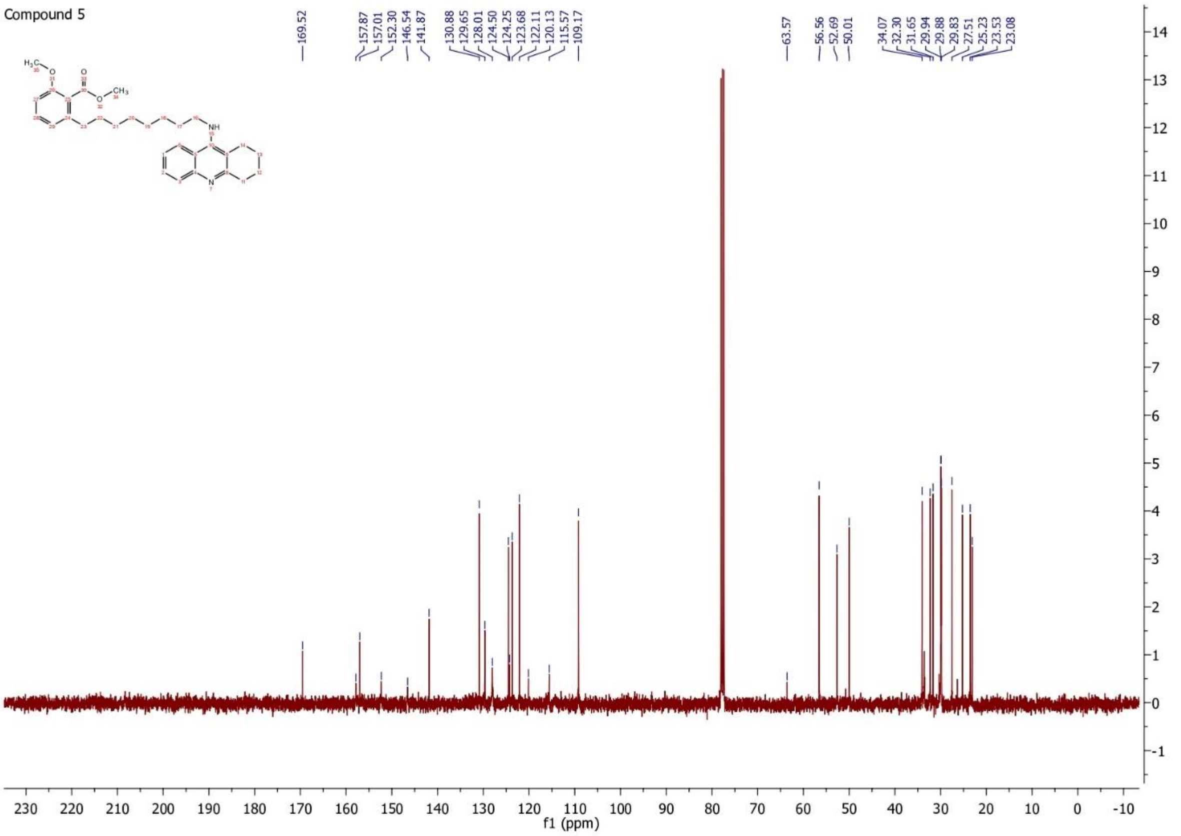

Compound 6

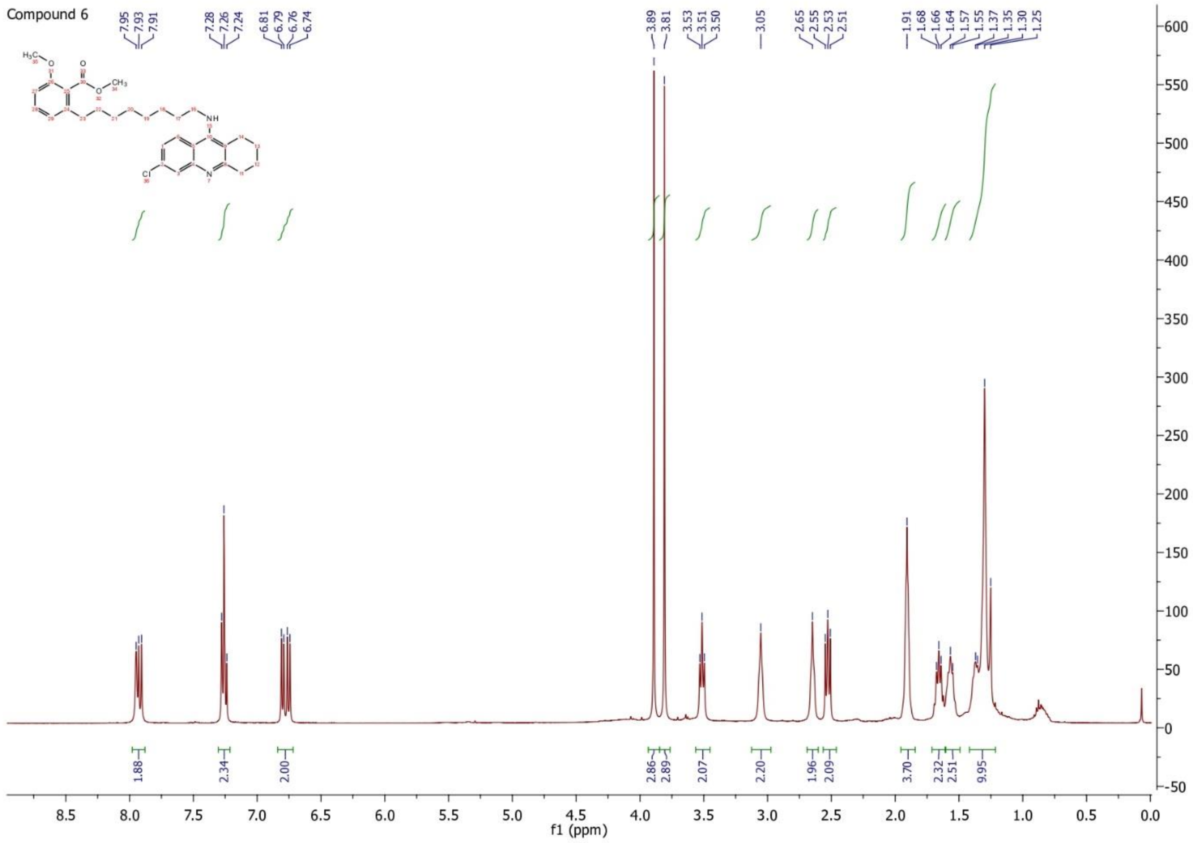

Compound 6
7.95 7.93 7.91 7.28 7.26 7.24 6.81 6.79 6.76 6.74 3.89 3.81 3.53 3.51 3.50 3.05 2.65 2.55 2.53 2.51 1.91 1.68 1.66 1.64 1.57 1.55 1.37 1.35 1.30 1.25
NH
Cl
N
1.88 2.34 2.00 2.86 2.89 2.07 2.20 1.96 2.09 3.70 2.32 2.51 9.95
f1 (ppm)

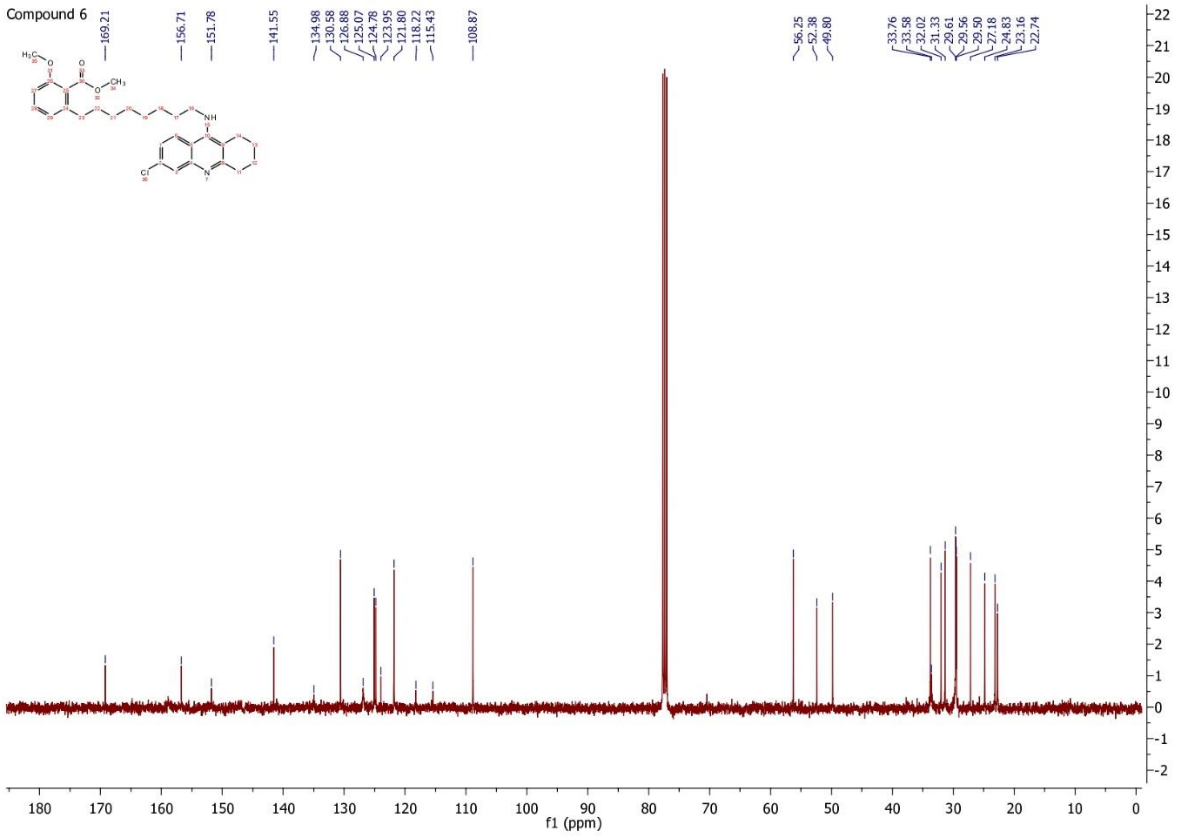

Compound 6
169.21 156.71 151.78 141.55 134.98 130.58 126.88 125.07 124.78 123.95 121.80 118.22 115.43 108.87 56.25 52.38 49.80 33.76 33.58 32.02 31.33 29.61 29.56 29.50 27.18 24.83 23.16 22.74
NH
Cl
N
f1 (ppm)

HRMS of 5 and 6.

Compound 5

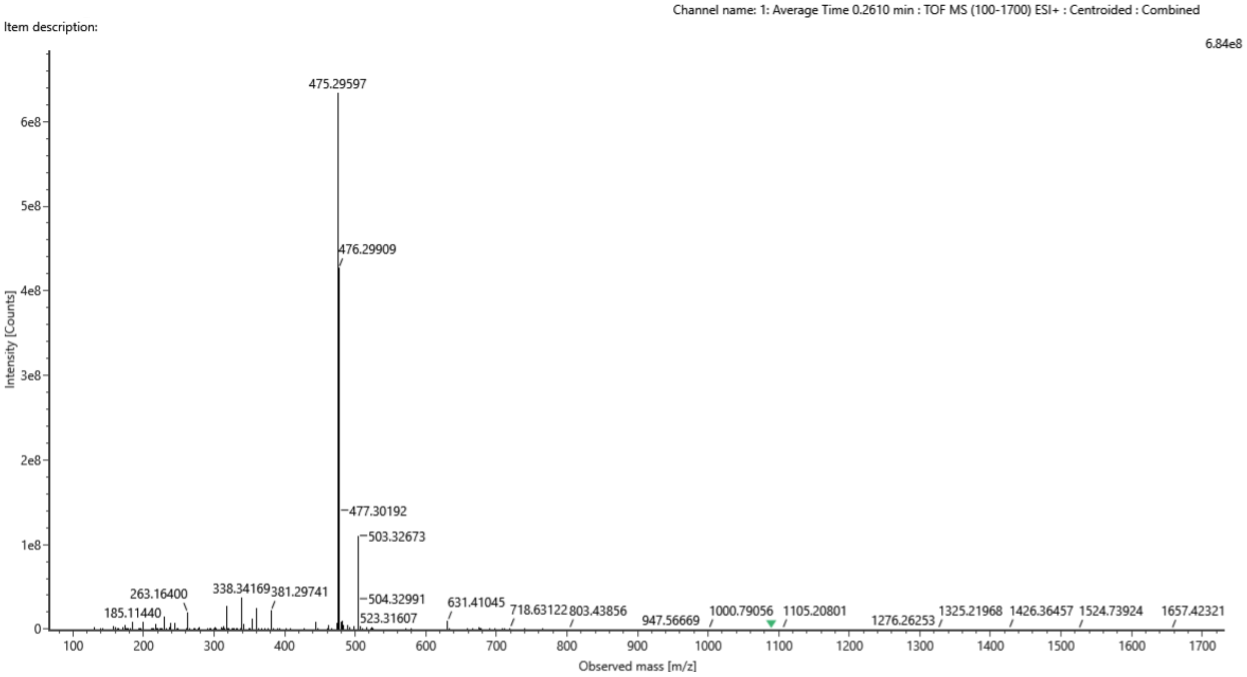

Compound 6

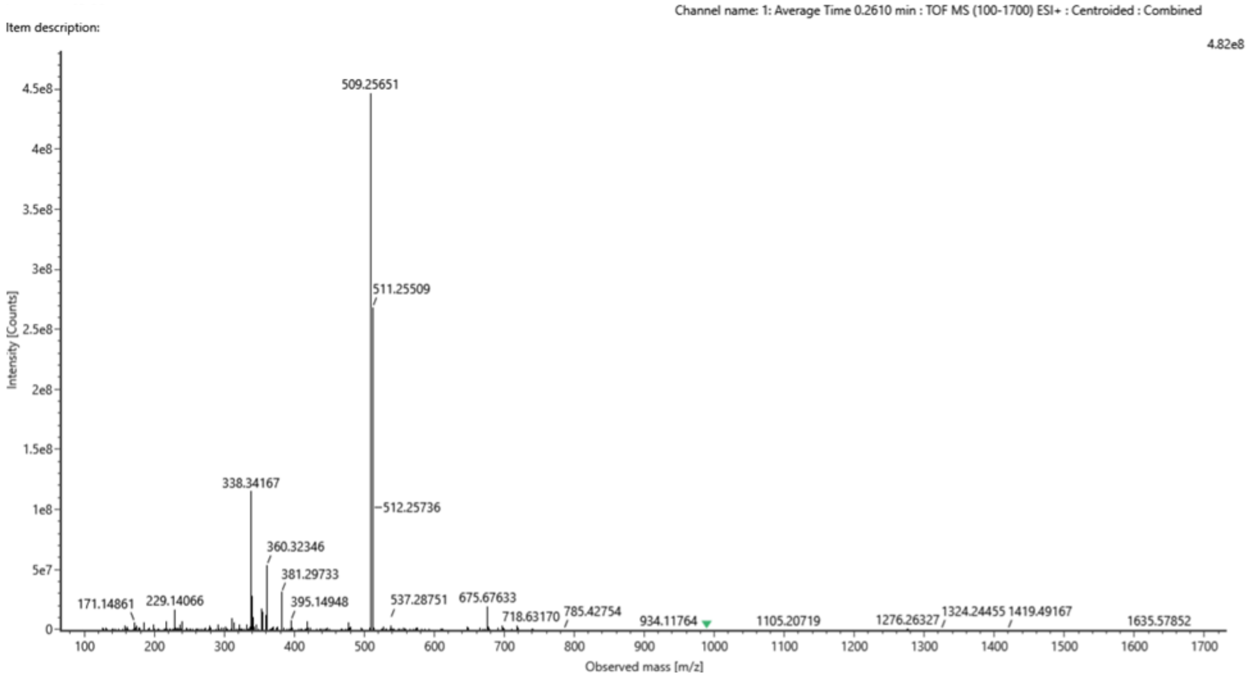

Data collection and processing, and general structural analysis. Crystals of the BChE-**5** complex (pdb code 7bgc) were obtained by cocrystallization of hBChE and **5**, knowing that a fast 5-min soaking of native crystals in the mother liquor containing 1 mM of ligand failed to form the complex. Diffraction data from two isomorphous crystals were merged and reflections up to 2.4 Å resolution were selected based on the CC1/2 criteria (>15%).[2] Data collection and refinement statistics are summarized in Table S1. After molecular replacement, we immediately identified two large disconnected positive peaks in the |Fo|-|Fc| map, the first one in the choline-binding pocket next to Trp82, the second one in the acyl-binding pocket extending from Trp231 to the catalytic serine. The elongated peak in the choline-binding pocket could be unambiguously modeled as the tacrine subpart of **5** thus suggesting that the second large peak corresponds to the dimethyl salicylate subpart. Iterative cycles of model building and refinement confirmed this interpretation (Figure 2A and 2B). The final overall structure is virtually identical to that of the original hBChE structure (pdb entry 1p0i) with all-atoms root mean square deviation (rmsd) of 0.51 Å. The N-glycan of the complex type linked to Asn57 is visible up to the terminal sialic acid, the latter forming a first H-bond with Asp87 of symmetry-related monomer of hBChE (3.0 Å) and second H-bond with the mainchain carbonyl of Pro429 of another symmetry-related monomer (2.5 Å). This highlights the importance of this particular glycosylation in the stabilization of crystalline recombinant hBChE. We observed a single change of side-chain orientation in the gorge region, in the acyl-binding pocket, with a 180° rotation around the Cβ-Cɣ bond of Leu286. This change is without significant consequence on the volume or shape of the pocket.

Table S2: **Data collection and refinement statistics.**

Calculated using Phenix. [3] R-work=Σ |fo| - |fc| / Σ |fo|, fo and fc are observed and calculated structure factors, R-free set uses about one thousand randomly chosen reflections. Statistics for the highest-resolution shell are shown in parentheses. Structure deposited in the Protein Data Bank under accession code **7bgc**.

Data collection	
X-ray source - beamline	SOLEIL - PROXIMA 2
Wavelength (Å)	0.9789
Resolution range (Å)<br>(highest-resolution shell)	54.59 - 2.4<br>(2.486 - 2.4)
Space group	I 4 2 2
Unit cell parameters (Å)	154.40 154.40 128.27 90 90 90
Total reflections	1505007 (139086)
Unique reflections	30505 (3009)
Multiplicity	49.3 (46.2)
Completeness (%)	99.83 (99.73)
Mean I/σ (I)	10.34 (0.40)
Wilson B-factor	80.40
R-merge	0.3724 (15.69)
R-meas	0.3763 (15.86)
R-pim	0.0535 (2.33)
CC1/2	0.999 (0.172)
CC*	1 (0.542)

Refinement	
Reflections used in refinement	30489 (3007)
Reflections used for R-free	1068 (105)
R-work	0.2031 (0.4709)
R-free	0.2471 (0.4687)
CC(work)	0.969 (0.429)
CC(free)	0.906 (0.392)
Number of non-hydrogen atoms	4613
macromolecules	4242
ligands	281
solvent	90
Protein residues	527
RMS bonds (Å)	0.009
RMS angles (Å)	1.11
Ramachandran favored (%)	89.33
Ramachandran allowed (%)	10.10
Ramachandran outliers (%)	0.57
Rotamer outliers (%)	0.22
Clashscore	14.19
Average B-factor (Å^2)	89.48
macromolecules (Å^2)	86.44
ligands (Å^2)	138.65
solvent (Å^2)	78.84
Number of TLS groups	1

Figure S1. Neurotoxicity of 5, 6, 9, 12-15 and 17 on SH-SY5Y cells

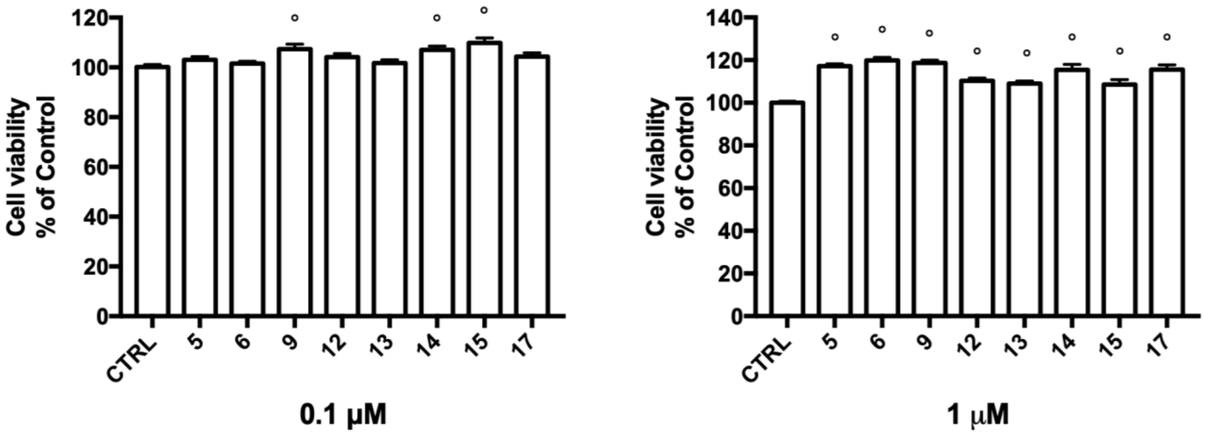

Figure S1. Cells were treated with increasing concentrations of the selected compounds (0.1-1 μM) for 24 h, and cell viability was evaluated by MTT assay. Each bar represents means ± SEM of at least four independent experiments. Data were analyzed by one-way ANOVA followed by Dunnett's test. °$p < 0.05$ compared to CTRL.

Figure S2. Cytotoxicity of 5, 6, 9, 12 - 15 and 17 on murine microglial BV-2 cells.

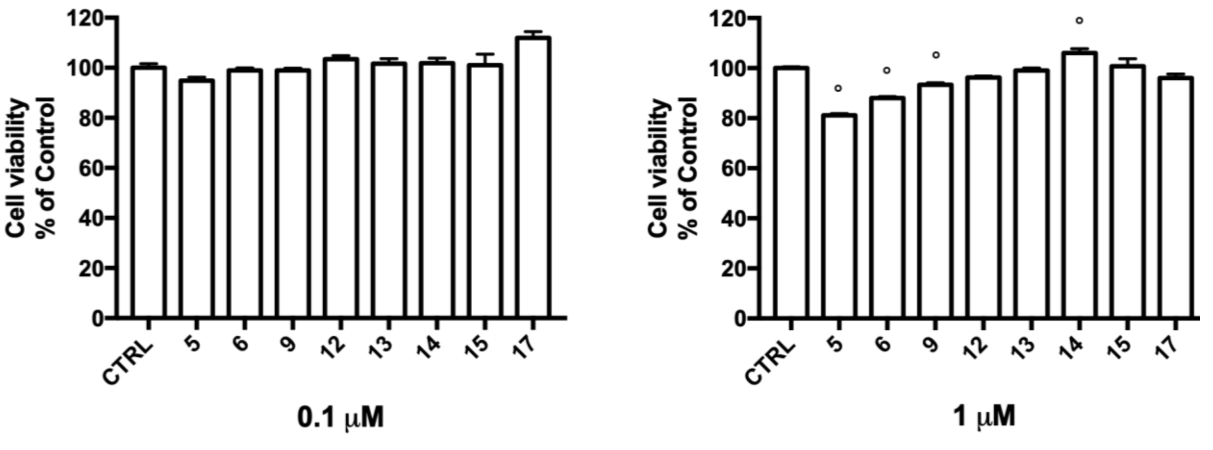

Figure S2. BV-2 cells were treated with increasing concentration of the selected compounds (0.1-1 μM) for 24 h, and cell viability was evaluated by MTT assay. Each bar represents means ± SEM of at least four independent experiments. Data were analyzed by one-way ANOVA followed by Dunnett's test. °$p < 0.05$ compared to CTRL

Table S3. BV-2 cell primers for real-time PCR.

Gene	Primer
GAPDH forward	5’ACCACAGTCCATGCCATCAC3’
GAPDH reverse	5’TCCACCACCCTGTTGCTGTA3’
IL-1β forward	5’GTTCCCATTAGACAACTGCACTACAG3’
IL-1β reverse	5’GTCGTTGCTTGGTTCTCCTTGTA3’
TNF-α forward	5’CCCCAAAGGGATGAGAAGTTC3’
TNF-α reverse	5’CCTCCACTTGGTGGTTTGCT3’
iNOS forward	5’CCTCCTCCACCCTACCAAGT3’
iNOS reverse	5’CACCCAAAGTGCTTCAGTCA3’
COX2 forward	5’TGGGGTGATGAGCAACTATT3’
COX2 reverse	5’AAGGAGCTCTGGGTCAAACT3’

Bibliography

1. Mezeiova, E.; Korabecny, J.; Sepsova, V.; Hrabinova, M.; Jost, P.; Muckova, L.; Kucera, T.; Dolezal, R.; Misik, J.; Spilovska, K.; Pham, N. L.; Pokrievkova, L.; Roh, J.; Jun, D.; Soukup, O.; Kaping, D.; Kuca, K., Development of 2-Methoxyhuprine as Novel Lead for Alzheimer's Disease Therapy. *Molecules* **2017,** *22* (8).

2. Karplus, P. A.; Diederichs, K., Assessing and maximizing data quality in macromolecular crystallography. *Curr. Opin. Struct. Biol.* **2015,** *34*, 60-8.

3. Adams, P. D.; Afonine, P. V.; Bunkoczi, G.; Chen, V. B.; Davis, I. W.; Echols, N.; Headd, J. J.; Hung, L. W.; Kapral, G. J.; Grosse-Kunstleve, R. W.; McCoy, A. J.; Moriarty, N. W.; Oeffner, R.; Read, R. J.; Richardson, D. C.; Richardson, J. S.; Terwilliger, T. C.; Zwart, P. H., PHENIX: a comprehensive Python-based system for macromolecular structure solution. *Acta Crystallogr. D Biol. Crystallogr.* **2010,** *66* (Pt 2), 213-21.
